# Association between triglyceride-glucose index and risk of incident diabetes: a secondary analysis based on a Chinese cohort study: TyG index and incident diabetes

**DOI:** 10.1186/s12944-020-01403-7

**Published:** 2020-11-08

**Authors:** Xiaoli Li, Guilong Li, Tiantian Cheng, Jing Liu, Guangyao Song, Huijuan Ma

**Affiliations:** 1 Department of Internal Medicine, Hebei Medical University, Shijiazhuang, 050017 Hebei China; 2 Department of Endocrinology and Metabolic Diseases, Hebei General Hospital, Shijiazhuang, 050051 Hebei China; 3 Department of Cardiology, Xingtai Third Hospital, Xingtai, 054000 Hebei China; 4 Clinical Medical College, North China University of Science and Technology, Tangshan, 063210 Hebei China; 5 Hebei Key Laboratory of Metabolic Diseases, Hebei General Hospital, Shijiazhuang, 050051 Hebei China

**Keywords:** Triglyceride-glucose index, Incident diabetes, Association, Nonlinearity, Insulin resistance, Cohort study, Chinese adults

## Abstract

**Background:**

Recent studies have suggested the triglyceride-glucose index (TyG index) may serve as a suitable substitute for insulin resistance. However, evidence for the relationship between TyG index and risk of diabetes remains limited. This study sought to explore the association of baseline TyG index with risk of developing diabetes in Chinese adults.

**Methods:**

This retrospective cohort study was conducted using data from the health screening program in China. A total of 201,298 non-diabetic individuals were included. TyG index was calculated as Ln [fasting plasma glucose (mg/dL) × fasting triglyceride level (mg/dL) / 2]. Diabetes was defined as fasting plasma glucose ≥126 mg/dL and/or self-reported diabetes. Cox proportion-hazard model was employed to evaluate the independent impact of baseline TyG index on future diabetes risk. Sensitivity and subgroup analyses were implemented to verify the reliability of results. Notably, data were downloaded from the DATADRYAD website, and used only for secondary analyses.

**Results:**

During an average follow-up of 3.12 years, among 201,298 individuals aged ≥20 years, 3389 subjects developed diabetes. After adjusting for potential confounders, elevated TyG index were independently correlated with greater risk of incident diabetes (hazard ratio (HR), 3.34; 95% confidence interval (CI), 3.11–3.60). Compared with the lowest quartile (Q1), increasing TyG index (Q2, Q3, and Q4) was related to increased HR estimates of incident diabetes [HR (95% CI), 1.83 (1.49–2.26); 3.29 (2.70–4.01), and 6.26 (5.15–7.60), respectively]. Moreover, a nonlinear relationship was observed between TyG index and risk of diabetes and the slope of the curve increased accompanying the rise of TyG index. Subgroup analysis revealed the positive association was stronger among subjects with age < 40 years, body mass index ≥18.5 kg/m^2^ and < 24 kg/m^2^, or systolic blood pressure < 140 mmHg, or in females.

**Conclusions:**

Elevated TyG index is independently correlated with increased risk of incident diabetes in Chinese adults, indicating it may represent a reliable predictor of diabetes in high-risk populations.

**Supplementary information:**

**Supplementary information** accompanies this paper at 10.1186/s12944-020-01403-7.

## Background

Diabetes has become an epidemic worldwide estimated to affect 439 million adults by 2030. It is a growing health problem imposing heavy financial burden on individuals and society [1–4]. To relieve this burden, public health strategies should focus on screening high-risk populations for incident diabetes, mainly for early prevention and appropriate intervention. Therefore, identification of a predictor that is easily measured, widely applicable, highly accurate, and especially easily intervened has important practical significance. Among various risk factors of diabetes, including metabolic, genetic and inflammatory risk factors, only some metabolic-related risk factors, such as dyslipidemia and high BMI, can be intervened through lifestyle changes [5–7].

Prospective studies have indicated insulin resistance (IR) remains the main pathogenesis of diabetes, which is present many years before diagnosis [8–10]. Clearly, accurate measurement of IR can improve the prediction of progression to diabetes. The hyperinsulinemic-euglycemic clamp (HIEC) technique continues to be the gold standard for quantitative IR [11], whereas it is costly and time-consuming to apply in clinical practice. The triglyceride-glucose index (TyG index), derived from triglyceride (TG) and fasting plasma glucose (FPG), was recommended as an alternative to IR in healthy subjects [12, 13] Several studies confirmed its accuracy for diagnosing IR, taking HIEC or homeostasis model assessment-IR (HOMA-IR) as reference standards [13–16]. Compared with insulin-based indices, the noninsulin-based TyG index is easily and inexpensively determined, which is advantageous in clinical and epidemiological research. Some studies revealed the TyG index was relevant with high risk of diabetes [17–20]. However, only one of the studies [17] was performed in China, with a relatively small sample size and individuals with normal body mass index (BMI), thereby limiting its generalizability. Therefore, the present study, based on a large cohort of 201,298 participants across 32 locations in 11 cities in China, sought to further explore the potential impact of baseline TyG index on future diabetes risk.

Remarkably, the original study was performed by Chen et al. [21], and the associated database was uploaded to the DATADRYAD website. The present report is a secondary analysis on the basis of the aforementioned database [21]. In the original study, the authors focused on the association of BMI with future diabetes risk [21]. In this secondary analysis, TyG index treated as independent variable, endpoint event and most covariates basically coincided with the original study.

## Methods

### Data source

Data were downloaded from the DATADRYAD website (www.datadryad.org), which allows others to freely obtain original data. In accordance with the Dryad Terms of Service, in this study, we refer to the following Dryad data package: Chen, Ying et al. (2018), data from: Association of body mass index and age with incident diabetes in Chinese adults: a population-based cohort study, Dataset, 10.5061/dryad.ft8750v. The following variables were involved in this database: sex, age, BMI, drinking, smoking, family history of diabetes, low density lipoprotein cholesterol (LDL-C), high density lipoprotein cholesterol (HDL-C), total cholesterol (TC), TG, FPG, serum creatinine (Scr), aspartate aminotransferase (AST), alanine aminotransferase (ALT), systolic blood pressure (SBP), diastolic blood pressure (DBP), FPG of final visit, incident diabetes at follow up and follow-up time. In the original paper [21], the authors declared that they have relinquished copyright and relevant ownership of the database. Thus, this database can be used for secondary analyses without violating the authors’ rights.

### Study population

Chen et al. performed the original study [21]. Here is a brief summary of their study protocol, the complete details of which are previously described [21]. The authors conducted a retrospective cohort study across 32 locations in 11 cities in China using data from a health screening project established by the Rich Healthcare Group. They recruited 685,277 participants who underwent at least two health checks between 2010 and 2016. Ultimately, 211,833 participants were enrolled according to eligibility criteria, and exclusive criteria included: (1) baseline height, weight, sex, or FPG were unavailable; (2) outliers of BMI (< 15 kg/m^2^ or > 55 kg/m^2^); (3) follow-up interval was less than 2 years; and (4) participants had diabetes at baseline or the status of diabetes was undefined at the deadline. Additionally, in the original article [21], Chen et al. declared the research was authorized by the Rich Healthcare Group Review Board. They only retrieved data retrospectively, and no subjects were required to participate in any part of the study, so the informed consent of participants were not involved in the study. In this report, some data were removed from the analysis cohort for further study: (1) missing TG values at baseline (*n* = 5747); and (2) extreme TG or FPG values (< mean – 3 standard deviations (SD) or > mean + 3SD) (*n* = 4789) [22]. In total, 201,298 subjects (109,236 males and 92,062 females) were included for analysis in this study.

### Measurement of the TyG index and other covariates

A detailed questionnaire was administered to obtain demographic characteristics, lifestyle, disease history, and medical history. Height measurement was accurate to 0.1 cm. When measuring weight (accurate to 0.1 kg), subjects were required to wear lightweight and no shoes. BMI was calculated as weight / height squared (kg/m^2^). Fasting venous blood was drawn to detect serum LDL-C, TG, TC, HDL-C and FPG values by an automatic biochemical analyzer (Beckman 5800). The TyG index was calculated as Ln [FPG (mg/dL) × fasting TG (mg/dL) / 2] [12]. Because this was a retrospective cohort study, observation bias was naturally reduced.

### Ascertainment of diabetes

Diabetes was defined according to FPG ≥ 126 mg/dL or self-reported diabetes. Ascertainment of diabetes depended on the date of diagnosis or the last visit.

### Statistical analysis

The missing values of other variables were first supplemented before statistical analysis. If the missing values were continuous variables (such as TC, LDL-C, ALT, AST, Scr, SBP and DBP), they were supplemented by the mean or median. When missing data were categorical variables (such as smoking and drinking status), they were treated as a set of categorical variables [23].

Data for qualitative variables are expressed as numbers (percentage), while data for quantitative variables are shown as median (25th–75th percentile) or mean ± SD. The statistical differences of percentage, median and mean among groups were verified by chi-square test, Kruskal-Wallis H test and one-way ANOVA, respectively. Cox proportional hazard model was used for evaluating the independent impact of TyG index on diabetes risk. In addition to the unadjusted model, results for the minor adjustment model (model I) and full adjustment model (model II) were presented. Taking TyG index as a categorical variable, sensitivity analysis was implemented to test the robustness of results. Additionally, a generalized additive model was employed to analyse the nonlinear relationship of TyG index with risk of diabetes. Subgroup analyses were implemented to further verify the robustness of the results. Furthermore, the likelihood ratio test was conducted to evaluate the interaction among subgroups. The Kaplan-Meier curve was used for generating cumulative event rates and the log-rank test was applied to compare outcome events distributions among groups.

Statistical analyses were carried on using R statistical software packages (http://www.r-project.org, The R Foundation) and EmpowerStats (http://www.empowerstats.com, X&Y Solutions, Inc., Boston, MA). A two-sided *P*-value < 0.05 was considered significant.

## Results

### Population selection

Of the 211,833 participants, 5746 were excluded for lack of baseline TG values, while 4789 were excluded because of extreme TG or FPG values, leaving 201,298 subjects for final data analysis.

### Baseline parameters of study population

A total of 201,298 subjects (54.3% male and 45.7% female) were involved in this study. Average age and BMI of the population were 42.08 ± 12.67 years and 23.19 ± 3.32 kg/m^2^, respectively. After an average follow-up of 3.12 years (SD, 0.94), 3389 participants were reported to have diabetes. The average TyG index was 8.35 ± 0.57, and baseline TyG index in diabetic patients was obviously higher than subjects without diabetes (8.90 ± 0.52 vs. 8.34 ± 0.57; *p* < 0.001). Table 1 displayed baseline parameters of the population by TyG index quartiles (< 7.93, 7.93–8.31, 8.31–8.73, ≥8.73). Except for HDL-C, which was not statistically different among the TyG quartiles, participants with higher TyG index generally had higher age, BMI, LDL-C, TC, ALT, AST, Scr, SBP, DBP, family history of diabetes, higher rates of smokers and drinkers.

**Table 1:** Baseline parameters of population (*N* = 201,298)

TyG index	Q1 (<7.93)	Q2 (≥7.93 to <8.31)	Q3 (≥8.31 to <8.73)	Q4 (≥8.73)	*P*-value
Participants	49,413	50,766	49,852	51,267	
Age (years, mean ± SD)	37.32 ± 9.93	40.48 ± 12.00	43.76 ± 13.26	46.61 ± 13.20	< 0.001
Sex, n (%).					< 0.001
Male	16,016 (32.41)	24,391 (48.05)	30,683 (61.55)	38,146 (74.41)	
Female	33,397 (67.59)	26,375 (51.95)	19,169 (38.45)	13,121 (25.59)	
BMI (kg/m^2^, mean ± SD)	21.26 ± 2.59	22.40 ± 2.95	23.71 ± 3.13	25.31 ± 3.11	< 0.001
SBP (mmHg, mean ± SD)	112.37 ± 14.02	116.50 ± 15.29	120.82 ± 16.07	125.62 ± 16.64	< 0.001
DBP (mmHg, mean ± SD)	69.98 ± 9.53	72.45 ± 10.09	75.10 ± 10.51	78.47 ± 10.92	< 0.001
FPG (mg/dL, mean ± SD)	83.49 ± 9.29	86.88 ± 9.40	89.61 ± 9.75	93.58 ± 10.48	< 0.001
TC (mg/dL, mean ± SD)	165.41 ± 28.64	175.71 ± 30.75	185.95 ± 32.89	198.45 ± 34.94	< 0.001
TG (mg/dL, mean ± SD)	51.00 ± 11.26	78.87 ± 12.03	113.51 ± 18.14	199.82 ± 58.97	< 0.001
LDL-C (mg/dL, mean ± SD)	79.28 ± 22.93	85.06 ± 27.52	90.81 ± 31.54	95.18 ± 34.56	< 0.001
HDL-C (mg/dL, mean ± SD)	53.17 ± 11.91	53.09 ± 11.82	53.08 ± 11.90	53.07 ± 11.79	0.769
ALT (IU/L, median (Q1-Q3)	13.90 (10.90–18.60)	16.00 (12.00–22.80)	19.05 (14.00–28.00)	25.40 (18.00–38.30)	< 0.001
AST (IU/L, mean ± SD)	21.74 ± 6.05	22.25 ± 6.41	22.95 ± 6.75	24.25 ± 8.22	< 0.001
Scr (mg/dL, mean ± SD)	0.73 ± 0.15	0.77 ± 0.16	0.81 ± 0.16	0.84 ± 0.16	< 0.001
Smoker					< 0.001
Now	1011 (2.05)	2096 (4.13)	3140 (6.30)	4824 (9.41)	
Once	304 (0.62)	536 (1.06)	732 (1.47)	847 (1.65)	
Never	10,591 (21.43)	11,372 (22.40)	10,978 (22.02)	10,556 (20.59)	
Not recorded	37,507 (75.90)	36,762 (72.41)	35,002 (70.21)	35,040 (68.35)	
Drinker					< 0.001
Now	108 (0.22)	225 (0.44)	329 (0.66)	581 (1.13)	
Once	1180 (2.39)	1831 (3.61)	2357 (4.73)	3128 (6.10)	
Never	10,618 (21.48)	11,948 (23.54)	12,164 (24.40)	12,518 (24.42)	
Not recorded	37,507 (75.91)	36,762 (72.41)	35,002 (70.21)	35,040 (68.35)	
Family history of diabetes, n (%)					< 0.001
No	48,526 (98.20)	49,708 (97.92)	48,804 (97.90)	50,158 (97.84)	
Yes	887 (1.80)	1058 (2.08)	1048 (2.10)	1109 (2.16)	

Values are presented as mean ± SD, median (Q1–Q3) or n (%)

*BMI* Body-mass index, *SBP* Systolic blood pressure, *DBP* Diastolic blood pressure, *FPG* Fasting plasma glucose, *TC* Total cholesterol, *TG* Triglyceride, *LDL-C* Low-density lipoprotein cholesterol, *HDL-C* High-density lipoprotein cholesterol, A*LT* Alanine aminotransferase, *AST* Aspartate transaminase, *Scr* Serum creatinine

### Univariate analysis

The univariate analyses of potential risk factors were presented in Table 2. The results revealed that these factors, including age, BMI, LDL-C, TC, TG, FPG, TyG index, ALT, AST, Scr, SBP, DBP, drinking, smoking and family history of diabetes, were positively related to future risk of diabetes, whereas HDL-C was not correlated with future risk. Besides, compared with males, females showed a lower risk of diabetes.

**Table 2:** The results of univariate analysis

	Statistics	HR (95% CI)	*P* value
Age (y)	42.08 ± 12.67	1.07 (1.06–1.07)	< 0.0001
Gender			
Male	109,236 (54.27%)	Ref	
Female	92,062 (45.73%)	0.51 (0.47–0.55)	< 0.0001
BMI (kg/m^2^)	23.19 ± 3.32	1.24 (1.23–1.25)	< 0.0001
SBP (mmHg)	118.88 ± 16.31	1.04 (1.04–1.04)	< 0.0001
DBP (mmHg)	74.03 ± 10.76	1.05 (1.04–1.05)	< 0.0001
LDL-C (mg/dL)	87.64 ± 30.11	1.01 (1.00–1.02)	< 0.0001
HDL-C (mg/dL)	53.10 ± 11.85	1.00 (1.00–1.01)	0.4881
TC (mg/dL)	181.51 ± 34.19	1.01 (1.01–1.01)	< 0.0001
TG (mg/dL)	111.41 ± 64.72	1.01 (1.01–1.01)	< 0.0001
FPG (mg/dL)	88.43 ± 10.43	1.15 (1.14–1.15)	< 0.0001
TyG index	8.35 ± 0.57	5.78 (5.44–6.14)	< 0.0001
Scr (mg/dL)	0.79 ± 0.17	2.87 (2.34, 3.52)	< 0.0001
ALT (U/L)	23.52 ± 19.95	1.01 (1.01–1.01)	< 0.0001
AST (U/L)	22.81 ± 6.98	1.01 (1.01–1.02)	< 0.0001
Smoker			
Now	11,071 (5.50%)	Ref	
Once	2419 (1.20%)	0.75 (0.56–1.00)	0.0506
Never	43,497 (21.61%)	0.46 (0.40–0.53)	< 0.0001
Not recorded	144,311 (71.69%)	0.63 (0.56–0.71)	< 0.0001
Drinker			
Now	1243 (0.62%)	Ref	
Once	8496 (4.22%)	0.48 (0.33–0.70)	0.0001
Never	47,248 (23.47%)	0.50 (0.35–0.70)	< 0.0001
Not recorded	144,311 (71.69%)	0.54 (0.39–0.76)	0.0003
Family history of diabetes			
No	197,196 (97.96%)	Ref	
Yes	4102 (2.04%)	1.74 (1.47–2.06)	< 0.0001

Data were expressed as mean ± SD or n (%)

*BMI* Body-mass index, *SBP* Systolic blood pressure, *DBP* Diastolic blood pressure, *TC* Total cholesterol, *TG* Triglyceride, *LDL-C* Low-density lipoprotein cholesterol, *HDL-C* High-density lipoprotein cholesterol, *FPG* Fasting plasma glucose, *TyG index* triglyceride-glucose index, *Scr* Serum creatinine, A*LT* Alanine aminotransferase, *AST* Aspartate transaminase

**Fig. 1:**
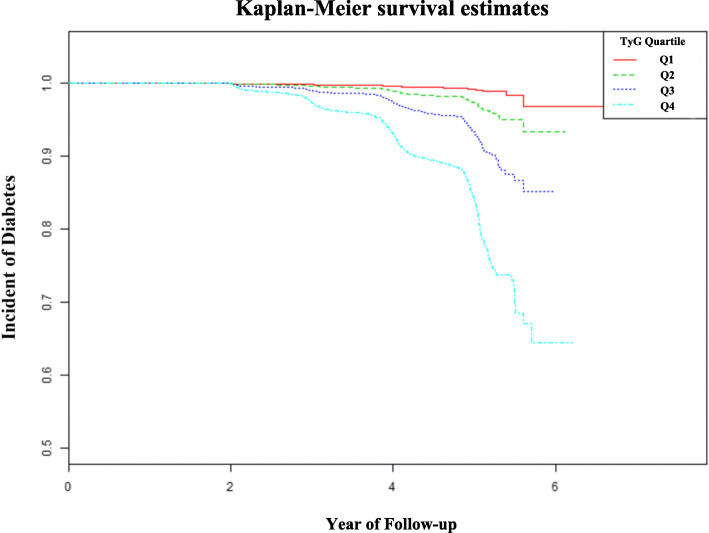
Kaplan-Meier analysis of future diabetes risk according to TyG index quartiles (log-rank, *P* < 0.0001)

As shown in Fig. 1, Kaplan-Meier curve revealed that the cumulative risk of incident diabetes was markedly different among the TyG index quartiles (log-rank test, *P* < 0.001) and increased gradually with increase of TyG index, resulting in maximum risk of diabetes in the highest quartile.

### Relationship between TyG index and future diabetes risk

As shown in Table 3, the effect of TyG index on risk of diabetes was assessed by cox proportional hazard model. In crude model, TyG index demonstrated a strongly positive association with future diabetes risk (HR, 5.78; 95% CI, 5.44–6.14). In model I (adjusted for sex, age and BMI), the positive correlation became relatively weaker (HR, 3.31; 95% CI, 3.09–3.55). In model II (further adjusted for TC, LDL-C, AST, ALT, Scr, DBP, SBP, drinking, smoking and family history of diabetes), the correlation did not change significantly compared with the minor adjustment model (HR, 3.34; 95% CI, 3.11–3.60).

**Table 3:** Relationship between TyG index and risk of diabetes

Outcomes	Crude model		Model I		Model II	
	HR (95% CI)	*P*	HR (95% CI)	*P*	HR (95% CI)	*P*
TyG index	5.78 (5.44–6.14)	< 0.0001	3.31 (3.09–3.55)	< 0.0001	3.34 (3.11–3.60)	< 0.0001
TyG (quartile)						
Q 1	Ref		Ref		Ref	
Q 2	2.88 (2.34–3.53)	< 0.0001	1.84 (1.50–2.27)	< 0.0001	1.83 (1.49–2.26)	0.0293
Q 3	7.45 (6.17–9.00)	< 0.0001	3.29 (2.70–3.99)	< 0.0001	3.29 (2.70–4.01)	0.0004
Q 4	19.94 (16.64–23.88)	< 0.0001	6.36 (5.26–7.70)	< 0.0001	6.26 (5.15–7.60)	< 0.0001
P for trend	< 0.0001		< 0.0001		< 0.0001	

Crude model: adjusted for none

Model I: adjusted for age, sex and BMI

Model II: adjusted for age, sex, BMI, LDL-C, TC, Scr, AST, ALT, SBP, DBP, drinking, smoking and family history of diabetes

To ensure the robustness of the results, TyG index was processed a categorical variable (quartiles) for sensitivity analysis. There was a graded and positive correlation of TyG index with future risk of diabetes. Compared with the lowest quartile (Q1), increasing TyG index (Q2, Q3, and Q4) was related to increased HR estimates of incident diabetes [HR (95% CI), 1.83 (1.49–2.26); 3.29 (2.70–4.01) and 6.26 (5.15–7.60), respectively].

### Nonlinear relationship

**Fig. 2:**
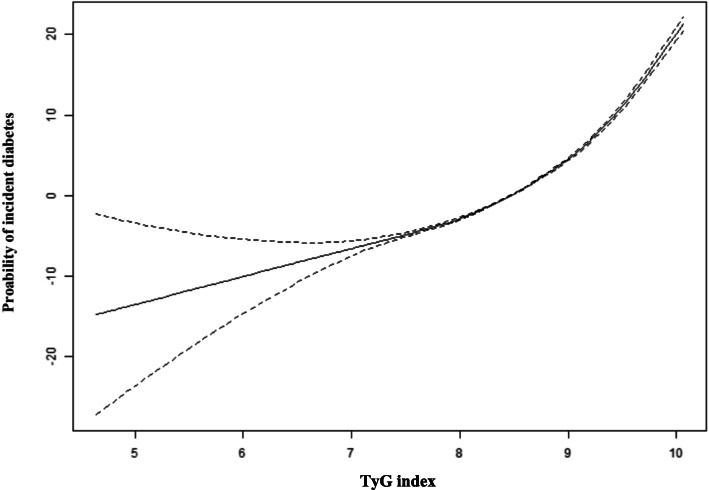
A nonlinear relationship of TyG index with risk of future diabetes. Note: the model was adjusted for sex, age, BMI, AST, ALT, LDL-C, TC, Scr, SBP, DBP, drinking, smoking and family history of diabetes

As shown in Fig. 2, after adjusting for sex, age, BMI, LDL-C, TC, ALT, AST, Scr, SBP, DBP, smoking, drinking, and family history of diabetes, a significant nonlinear relationship was found between TyG index and risk of future diabetes (*P* < 0.001), and the slope of the curve showed an upward tendency with the increase of TyG index.

### Subgroup analysis

To further investigate the impact of other risk factors on the correlation of TyG index with future diabetes risk, subgroup analyses were carried on according to the following stratification variables: sex, age, BMI, DBP, SBP, smoking, drinking and family history of diabetes. The results of subgroup analyses and interactions were summarized in Table 4. The additive interactions between TyG index and diabetes risk were observed in sex, age, BMI, and SBP (*P*-value for interaction < 0.05). Stronger correlations were found in participants with age < 40 years, BMI ≥ 18.5 kg/m^2^ and < 24 kg/m^2^, or SBP < 140 mmHg, or in females. However, significant interactions were not found in DBP, smoking, drinking, or family history of diabetes.

**Table 4:** Effect of magnitude of TyG index on diabetes risk stratified by subgroups

Characteristics	No. of participants	HR (95%CI)	*P*-value	*P* for interaction
Age (year)				< 0.0001
< 40	106,447	4.53 (3.76–5.45)	< 0.0001	
> =40, < 60	71,176	3.54 (3.19–3.93)	< 0.0001	
> =60	23,675	2.67 (2.37–3.00)	< 0.0001	
Sex				0.0150
Male	109,236	3.16 (2.90–3.45)	< 0.0001	
Female	92,062	3.84 (3.37–4.37)	< 0.0001	
BMI (kg/m^2^)				0.0075
< 18.5	11,593	3.64 (1.53–8.64)	0.0034	
> =18.5, < 24	112,241	4.13 (3.62–4.71)	< 0.0001	
> =24, < 28	60,886	3.22 (2.90–3.58)	< 0.0001	
> =28, < 32	14,388	3.11 (2.64, 3.68)	< 0.0001	
> =32	2190	3.36 (2.33, 4.85)	< 0.0001	
SBP (mmHg)				< 0.0001
< 140	181,383	3.48 (3.19–3.79)	< 0.0001	
> =140	19,915	2.89 (2.53–3.29)	< 0.0001	
DBP (mmHg)				0.9984
< 90	185,636	3.29 (3.04–3.56)	< 0.0001	
> =90	15,661	3.43 (2.90–4.05)	< 0.0001	
Smoker				0.6979
Now	11,071	3.03 (2.36–3.90)	< 0.0001	
Once	2419	4.27 (2.30–7.91)	< 0.0001	
Never	43,497	3.40 (2.84–4.08)	< 0.0001	
Not recorded	144,311	3.39 (3.13–3.69)	< 0.0001	
Drinker				0.2174
Now	1243	5.31 (2.34–12.05)	< 0.0001	
Once	8496	3.65 (2.52–5.28)	< 0.0001	
Never	47,248	3.33 (2.84–3.90)	< 0.0001	
Not recorded	144,311	3.39 (3.13–3.69)	< 0.0001	
Family history of diabetes				0.1175
No	197,196	3.39 (3.15–3.65)	< 0.0001	
Yes	4102	3.07 (2.10–4.50)	< 0.0001	

**Note 1:** the model was adjusted for sex, age, BMI, LDL-C, TC, Scr, ALT, AST, SBP, DBP, drinking, smoking and family history of diabetes

**Note 2:** the model was adjusted for all above variables except the corresponding stratification variable

## Discussion

This retrospective cohort study revealed that raised TyG index was independently correlated with greater risk of developing diabetes among apparently healthy adults in China (HR, 3.34; 95% CI, 3.11–3.60). Besides, a significant nonlinear relationship was observed and showed the risk of diabetes tend to ascend with increase of TyG index. Compared with the lowest quartile, individuals with the top quartile of TyG index demonstrated a sixfold greater risk of developing diabetes (Q4 vs. Q1; adjusted HR 6.26, 95% CI 5.15–7.60). Additionally, the results of subgroup analysis revealed this correlation existed regardless of participants being male or female, younger or older, or obese or nonobese, suggesting our results were robust and the TyG index was suitable for a wide range of subjects. Moreover, stronger associations were observed in participants with age < 40 years, BMI ≥ 18.5 kg/m^2^ and < 24 kg/m^2^, or SBP < 140 mmHg, or in females.

The TyG index, derived from FPG and TG, was proven as a marker of IR in many epidemiological studies [12–16, 24]. Compared with HIEC, the TyG index had high sensitivity (96.5%) as well as good specificity (85.0%) for diagnosing IR in a Mexican population [13], and was a more accurate predictor than HOMA-IR in a Brazilian study [14]. Moreover, consistent with this study, several studies suggested that high TyG index was relevant to future risk of T2DM in different races, as shown in reports from Korea, Singapore, and Europe [18–20, 25]. Similar results were observed in another Chinese cohort study [17] and the trend of nonlinear relationship of TyG index with diabetes risk was generally consistent with our study. However, the study only included 5706 subjects with normal BMI and was conducted in rural areas. Therefore, its generalizability is relatively limited. This study was based on a large cohort of 201,298 apparently healthy adults across 32 sites in 11 cities, and is clearly applicable to a relatively wide range of individuals, and provides a stronger basis for clinical promotion and application. Similarly, the risk of diabetes in a Singaporean population elevated progressively across TyG index quartiles (Q) from Q1 to Q4 (Q4 vs. Q1; adjusted HR 5.30, 95% CI 2.21–12.71) [20]. However, potential confounders, such as serum lipid index (LDL-C and TC), drinking, smoking and family history of diabetes, were not sufficiently adjusted, and were notablely relevant to high risk of diabetes [26–31]. Fortunately, these confounding factors were taken into consideration in this study to avoid potential effects on the results.

Islet β-cell dysfunction and IR remain the core pathological trait of T2DM [32]. Interestingly, the TyG index, besides being a substitute of IR, is associated with susceptibility of β-cells to glucotoxicity and lipotoxicity. Pancreatic β-cells show weak antioxidant enzyme defense, and oxidative stress has been proved to be an important feature for the pathogenesis and development of type 2 diabetes (T2DM) [33–35]. Besides, some indirect evidence also suggested that appropriate antioxidant supplementation can regulate lipid metabolism and improve insulin sensitivity [36–38]. Evidence suggested that elevated glucose levels can induce reactive oxygen species generation on islet β-cells, which in turn cause oxidative stress and β-cells dysfunction, and then lead to IR and T2DM [33–35]. Other studies revealed that long-term high free fatty acid content was related to prolonged exposure of TG in pancreatic islets, which may impair pancreatic β-cell function [39–41]. Furthermore, glycotoxicity and lipotoxicity were interactive rather than independent adverse effects on pancreatic β-cell [42–44]. Long term exposure of pancreatic beta cells to high fatty acids concentrations could result in impaired glucose-induced insulin secretion [45, 46] and increased β-cell death [47]. An intervention study confirmed that patients with impaired glucose metabolism had improved insulin secretion ability after being treated with n-3 fatty acids [48]. Besides, IR is largely attributable to the impairment of insulin-stimulated glucose absorption into skeletal muscle. When TG levels in peripheral blood and skeletal muscle were significantly increased, glucose metabolism in skeletal muscle would be impaired [49]. Therefore, to a certain extent, the TyG index reflects muscle IR [50].

Subgroup analysis and exploration of interactions is critical for clinical research, to better understand the actual relationships between independent variables and dependent variables [51]. Unfortunately, the related studies described above only used sex, and/or age as stratification factors for subgroup analyses [17–19], and no interactions were observed, which may hinder our understanding of the real association of TyG index with future diabetes risk. In this study, these factors, including BMI, sex, age, DBP, SBP, drinking, smoking and family history of diabetes, were taken as stratified variables, and stronger associations were observed in participants with age < 40 years, BMI ≥ 18.5 kg/m^2^ and < 24 kg/m^2^, or SBP < 140 mmHg, or in females. This association was particularly obvious in females, and was consistent with the cohort study by Zhang et al. [17]. This may be because serum lipids in female hepatocytes were higher than that in male hepatocytes under fasting and glucose lipid loading [52, 53]. In clinical practice, lean and obese individuals are not homogeneous, and obese individuals are generally considered more likely to develop diabetes. However, in the subgroup analysis based on BMI, regardless of lean individuals (BMI < 18.5 kg/m^2^) or obese individuals (BMI > 32 kg/m^2^), TyG index demonstrated a strongly positive association with future diabetes risk in this study [(BMI < 18.5 kg/m^2^, HR (95% CI), 3.64 (1.53–8.64); and BMI ≥ 32 kg/m^2^, HR (95% CI), 3.36 (2.33–4.85); respectively)]. The possible explanation for this result was that the mechanism of TyG index mediating diabetes in different BMI populations might be different. High TyG index, in obese individuals, may increase the risk of diabetes mainly by increasing insulin resistance, while in lean individuals mainly by damaging β-cells through glycotoxicity and lipotoxicity. On the whole, based on subgroup analysis, the TyG index appeared to be more sensitive for predicting risk of diabetes in younger individuals and those with normal BMI or SBP, suggesting it may be promising for screening risk of future diabetes, especially in individuals without high-risk factors such as hypertension, obesity and older age.

### Study strengths and limitations

This study had several advantages. First, it was based on a large sample cohort study with broad age spectrum. Therefore, there were sufficient subjects for analysis to guarantee dependability and robustness of results. Furthermore, the results are applicable to a relatively wide range of individuals. Other similar cohort studies had relatively small sample sizes and populations that tended to be older. Second, taking TyG index as continuous variable and categorical variable respectively, sensitivity analysis and trend test were carried out to improve the reliability of results and avoid the contingency in data analysis. Finally, subgroup analyse and interaction test were conducted to further prove the dependability of the results and identify potential interactions with other variables. This study also had limitations. Firstly, diabetes was diagnosed depending on FPG ≥ 126 mg/dL or self-reported diabetes, rather than by glycosylated hemoglobin or 2-h oral glucose tolerance test, which was probably underestimated. Secondly, this study did not distinguish between types of diabetes. However, these findings may be more applicable to T2DM, which accounts for approximately 90–95% of all diabetes cases. Thirdly, data on fasting insulin levels and glycosylated hemoglobin are not available in the database, so it is impossible to compare the accuracy of predicting diabetes risk between TyG and HOMA-IR or glycosylated hemoglobin. Fourthly, as this large cohort study was conducted in China, these findings can not be generalized to other races and certain populations, such as children and pregnant women. Finally, the present report was a secondary analysis on the basis of existing database, and although numerous confounding factors had been adjusted, some variables not included in the database, such as physical activity, dietary factors, and lipid-lowering agents, failed to be adjusted. Therefore, potential effects of these residual confounding factors on the results could not be ignored.

## Conclusions

This study manifested that elevated TyG index was independently correlated with increased risk of developing diabetes in Chinese adults. Besides, these findings expand the current knowledge that the TyG index seems to be more sensitive for predicting risk of diabetes in women, younger individuals, and those with normal BMI or SBP. The TyG index may therefore represent a reliable predictor for screening individuals at early diabetes risk, especially in people without high-risk factors such as older age, hypertension and obesity.

## Supplementary information

**Additional file 1.**

**Additional file 2.**

**Additional file 3.**

## Data Availability

Data can be downloaded from ‘DATADRYAD’ database (www.Datadryad.org).

## Abbreviations

TyG index: Triglyceride-glucose index
BMI: Body mass index
FPG: Fasting plasma glucose
TG: Triglyceride
TC: Total cholesterol
HDL-C: high density lipoprotein cholesterol
LDL-C: low density lipoprotein cholesterol
Scr: Serum creatinine
ALT: Alanine aminotransferase
AST: Aspartate aminotransferase
DBP: Diastolic blood pressure
SBP: Systolic blood pressure
IR: Insulin resistance
