# Correction to: Association between triglyceride-glucose index and risk of incident diabetes: a secondary analysis based on a Chinese cohort study

**DOI:** 10.1186/s12944-021-01432-w

**Published:** 2021-01-30

**Authors:** Xiaoli Li, Guilong Li, Tiantian Cheng, Jing Liu, Guangyao Song, Huijuan Ma

**Affiliations:** 1 Department of Internal Medicine, Hebei Medical University, Shijiazhuang, 050017 Hebei China; 2 Department of Endocrinology and Metabolic Diseases, Hebei General Hospital, Shijiazhuang, 050051 Hebei China; 3 Department ofCardiology, Xingtai Third Hospital, Xingtai, 054000 Hebei China; 4 Clinical Medical College, North China University of Science and Technology, Tangshan, 063210 Hebei China; 5 Hebei Key Laboratory of Metabolic Diseases, Hebei General Hospital, Shijiazhuang, 050051 Hebei China

**Correction to: Lipids Health Dis 19, 236 (2020)**

**https://doi.org/10.1186/s12944-020-01403-7**

Following publication of the original article [1], the authors noticed a phrase under title “TyG index and incident diabetes” that should be deleted as it is a short running title that has no relation to the main title. The original article has been updated.
