# Supplementary material for: Association between triglyceride-glucose index and risk of incident diabetes: a secondary analysis based on a Chinese cohort study: TyG index and incident diabetes
Source: Lipids Health Dis. 2020 Nov 8;19:236. doi: 10.1186/s12944-020-01403-7 (PMC7649000; doi:10.1186/s12944-020-01403-7)
Supplement: Supplementary file 1 — Additional file 1. [file 12944_2020_1403_MOESM1_ESM.pdf]

Association of triglyceride-glucose index and the risk of incident diabetes: a secondary analysis based on a Chinese cohort study

By Xiaoli Li

WORD COUNT 5999
TIME SUBMITTED 30-AUG-2020 12:03AM
PAPER ID 62641344

Association between triglyceride-glucose index and risk of incident diabetes: a secondary analysis based on a Chinese cohort study

Subheadings: TyG index and incident diabetes

Xiaoli Li[1,2], Guilong Li[3], Tiantian Cheng[2], Jing Liu[2], Guangyao Song[1,2] and Huijuan Ma[1,4*]

[1]Department of Internal Medicine, Hebei Medical University, Shijiazhuang 050017, Hebei, China

[2]Department of Endocrinology and Metabolic Diseases, Hebei General Hospital, Shijiazhuang 050051, Hebei, China

[3]Department of Cardiology, Xingtai Third Hospital, Xingtai 054000, Hebei, China

050051, Hebei, China

[4]Hebei Key Laboratory of Metabolic Diseases, Hebei General Hospital, Shijiazhuang 050051, Hebei, China

***Corresponding author:**

Huijuan Ma, Email: huijuanma76@163.com

Abstract

Background: Recent studies have suggested the triglyceride-glucose index (TyG index) may serve as a suitable substitute for insulin resistance. However, evidence for the relationship between TyG index and risk of diabetes remains limited. This study sought to explore the association of baseline TyG index with risk of developing diabetes in Chinese adults.

Methods: This retrospective cohort study was conducted using data from the health screening program in China. A total of 201,298 non-diabetic individuals were included. TyG index was calculated as Ln [fasting plasma glucose (mg/dl) × fasting triglyceride level (mg/dl)/2]. Diabetes was defined as fasting plasma glucose ≥7.0 mmol/L and/or self-reported diabetes. Cox proportion-hazard model was employed to evaluate the independent impact of baseline TyG index on future diabetes risk. Sensitivity and subgroup analyses were implemented to verify the reliability of results. Notably, data were downloaded from the DATADRYAD website, and used only for secondary analyses.

Results: During an average follow-up of 3.12 years, among 201,298 individuals aged ≥ 20 years, 3,389 subjects developed diabetes. After adjusting for potential confounders, elevated TyG index were independently correlated with greater risk of incident diabetes (hazard ratio (HR), 3.34; 95% confidence interval (CI), 3.11–3.60). Compared with the lowest quartile (Q1), increasing TyG index (Q2, Q3, and Q4) was related to increased HR estimates of incident diabetes [HR (95% CI), 1.83 (1.49–2.26); 3.29 (2.70–4.01),

and 6.26 (5.15–7.6), respectively]. Moreover, a nonlinear relationship was observed between TyG index and risk of diabetes and the slope of the curve increased accompanying the rise of TyG index. Subgroup analysis revealed the positive association was stronger among subjects with age< 40 years, body mass index ≥18.5 kg/m^2 and < 24 kg/m^2, or systolic blood pressure <140 mmHg, or in females.

Conclusions: Elevated TyG index is independently correlated with increased risk of incident diabetes in Chinese adults, indicating it may represent a reliable predictor of diabetes in high-risk populations.

Keywords: Triglyceride-glucose index, Incident diabetes, Cohort study, Chinese adults

Background

Diabetes has become an epidemic worldwide estimated to affect 439 million adults by 2030. It is a growing health problem imposing heavy financial burden on individuals and society [1–4]. To relieve this burden, public health strategies should focus on screening high-risk populations for incident diabetes, mainly for early prevention and appropriate intervention. Therefore, identification of a predictor that is easily measured, widely applicable, and highly accurate has important practical significance.

Prospective studies have indicated insulin resistance (IR) remains the main pathogenesis of diabetes, which is present many years before diagnosis [5-7]. Clearly, accurate measurement of IR can improve the prediction of progression to diabetes. The hyperinsulinemic-euglycemic clamp (HIEC) technique continues to be the gold standard for quantitative IR [8], whereas it is costly and time-consuming to apply in clinical practice. 19 The triglyceride-glucose index (TyG index), derived from triglyceride (TG) and fasting plasma glucose (FPG), was recommended as an alternative to IR in healthy subjects [9, 10] Several studies confirmed its accuracy for diagnosing IR, taking HIEC or homeostasis model assessment-IR (HOMA-IR) as reference standards [10-13]. Compared with insulin-based indices, the noninsulin-based TyG index is easily and inexpensively determined, which is advantageous in clinical and epidemiological research. Some studies revealed the TyG index was relevant with high risk of diabetes mellitus [14-17]. However, only one of the studies [14] was performed in China, with relatively small sample size and individuals with normal body mass index (BMI),

thereby limiting its generalizability. Therefore, the present study, based on a large cohort of 201,298 participants across 32 locations in 11 cities in China, sought to further explore the potential impact of baseline TyG index on future diabetes risk.

Remarkably, the original study was performed by Chen et al. [18], and associated database was uploaded to the DATADRYAD website. The present report is a secondary analysis on the basis of the aforementioned database [18]. In the original study, the authors focused on the association of BMI with future diabetes risk [18]. In this secondary analysis, TyG index treated as independent variable, endpoint event and most covariates basically coincided with the original study.

Methods

Data source

Data were downloaded from the DATADRYAD website (www.datadryad.org), which allows others to freely obtain original data. In accordance with the Dryad Terms of Service, in this study, we refer to the following Dryad data package: Chen, Ying et al. (2018), Data from: Association of body mass index and age with incident diabetes in Chinese adults: a population-based cohort study, Dataset, https://doi.org/10.5061/dryad.ft8750v. The following variables were involved in this database: sex, age, BMI, drinking, smoking, family history of diabetes, low density lipoprotein cholesterol (LDL-C), high density lipoprotein cholesterol (HDL-C), total cholesterol (TC), TG, FPG, serum creatinine (Scr), aspartate aminotransferase (AST), alanine aminotransferase (ALT), systolic blood pressure (SBP), diastolic blood pressure

(DBP), FPG of final visit, incident diabetes at follow up and follow-up time. In the original paper [18], the authors declare that they have relinquished copyright and relevant ownership of the database. Thus, this database can be used for secondary analyses without violating the authors' rights.

Study population

Chen et al. performed the original study [18]. Here, we provide a brief summary of their study protocol, the complete details of which are previously described [18]. The authors conducted a retrospective cohort study across 32 locations in 11 cities in China using data from a health screening project established by the Rich Healthcare Group. They recruited 685,277 participants who underwent at least two health checks between 2010 and 2016. Ultimately, 211,833 participants were enrolled according to eligibility criteria, and exclusive criteria included: (1) baseline height, weight, sex, or FPG were unavailable; (2) outliers of BMI (<15 kg/m^2 or >55 kg/m^2); (3) follow-up interval was less than 2 years; and (4) participants had diabetes at baseline or the status of diabetes was undefined at the deadline. Additionally, in the original article [18], Chen et al. declared the research was authorized by the Rich Healthcare Group Review Board. They only retrieved data retrospectively, and no subjects were required to participate in any part of the study, so the informed consent of participants were not involved in the study. In this report, we removed some data from the analysis cohort for further study: (1) missing TG values at baseline (n = 5,747); and (2) extreme TG or FPG values (< mean – 3 standard deviations (SD) or > mean + 3SD) (n = 4,789) [18]. In total, 201,298

subjects (109,236 males and 92,062 females) were included for analysis in this study.

Measurement of the TyG index and other covariates

A detailed questionnaire was administered to obtain demographic characteristics, lifestyle, disease history, and medical history. Height measurement was accurate to 0.1 cm. When measuring weight (accurate to 0.1 kg), subjects were required to wear lightweight and no shoes. 26 BMI was calculated as weight / height squared (kg/m2). Fasting venous blood was drawn to detect serum LDL-C, TG, TC, HDL-C and FPG values by an automatic biochemical analyzer (Beckman 5800). 14 The TyG index was calculated as Ln [FPG (mg/dl) × fasting TG (mg/dl)/2] [9]. Because 22 this was a retrospective cohort study, observation bias was naturally reduced.

Ascertainment of diabetes

Diabetes was defined according to FPG ≥ 18 7.0 mmol/L or self-reported diabetes. Ascertainment of diabetes depended on the date of diagnosis or the last visit.

Statistical analysis

We first dealt with the missing values before statistical analysis. If the missing values were continuous variables (such as TC, LDL-C, ALT, AST, Scr, SBP and DBP), they were supplemented by the mean or median. When missing data were categorical variables (such as smoking and drinking status), they were treated as a set of categorical variables [20].

Data for 35 qualitative variables are expressed as numbers (percentage), while data for quantitative variables are shown as median (25th-75th percentile) or mean ± SD. The

statistical differences of percentage, median and mean among groups were verified by chi-square test, Kruskal-Wallis H test and one-way ANOVA, respectively. Cox proportional hazard model was used for evaluating the independent impact of TyG index on diabetes risk. In addition to the unadjusted model, results for the minor adjustment model (model I) and full adjustment model (model II) were presented. Taking TyG index as a categorical variable, sensitivity analysis was implemented to test the robustness of results. Additionally, generalized additive model was employed to analyse the nonlinear relationship of TyG index with risk of diabetes. Subgroup analyses were implemented to further verify the robustness of the results. Furthermore, the likelihood ratio test was conducted to evaluate the interaction among subgroups. The Kaplan-Meier curve was used for generating cumulative event rates and the log-rank test was applied to compare outcome events distributions among groups.

Statistical analyses were carried on using R statistical software packages (http://www.r-project.org, The R Foundation) and EmpowerStats (http://www.empowerstats.com, X&Y Solutions, Inc., Boston, MA). A two-sided p-value < 0.05 was considered significant.

Results

Population selection

Of the 211,833 participants, 5,746 were excluded for lack of baseline TG values, while 4,789 were excluded because of extreme TG or FPG values, leaving 201,298 subjects for final data analysis.

Baseline parameters of study population

A total of 201,298 subjects (54.3% male and 45.7% female) were involved in this study. Average age and BMI of the population were 42.08 ± 12.67 years and 23.19 ± 3.32 kg/m^2, respectively. After an average follow-up of 3.12 years (SD, 0.94), 3,389 participants were reported to have diabetes. Average TyG index was 8.35 ± 0.57, and the TyG index in diabetic patients was obviously higher than subjects without diabetes (8.90 ± 0.52 vs. 8.34 ± 0.57; $p<0.001$). Table 1 displayed baseline parameters of the population by TyG index quartiles (<7.93, 7.93-8.31, 8.31-8.73, ≥8.73). Except for HDL-C, which was not statistically different among the TyG quartiles, participants with higher TyG index generally had higher age, BMI, LDL-C, TC, ALT, AST, Scr, SBP, DBP, and higher rates of smokers, drinkers and family history of diabetes.

Univariate analysis

The univariant analysis of potential risk factors were presented in Table 2. The results revealed that these factors, including age, BMI, LDL-C, TC, TG, FPG, TyG index, ALT, AST, Scr, SBP, DBP, drinking, smoking and family history of diabetes, were positively related to future risk of diabetes, whereas HDL-C was not correlated with future diabetes risk. Besides, compared with males, females showed a lower risk of diabetes.

As shown in Figure 1, Kaplan-Meier curve revealed that the cumulative risk of incident diabetes was markedly different among the TyG index quartiles (log-rank test, $p<0.001$) and increased gradually with increase of TyG index, resulting in maximum risk of diabetes in the highest quartile.

Table 1. Baseline parameters of population (N = 201,298)

TyG index	Q1 (＜7.93)	Q2 (≥7.93 to ＜8.31)	Q3 (≥8.31 to ＜8.73)	Q4 (≥8.73)	P-value
Participants	49,413	50,766	49,852	51,267	
Age (years, mean ± SD)	37.32 ± 9.93	40.48 ± 12.00	43.76 ± 13.26	46.61 ± 13.20	<0.001
Sex, n (%).					<0.001
Male	16,016 (32.41)	24,391 (48.05)	30,683 (61.55)	38,146 (74.41)	
Female	33,397 (67.59)	26,375 (51.95)	19,169 (38.45)	13,121 (25.59)	
BMI (kg/m^2, mean ± SD)	21.26 ± 2.59	22.40 ± 2.95	23.71 ± 3.13	25.31 ± 3.11	<0.001
SBP (mmHg, mean ± SD)	112.37 ± 14.02	116.50 ± 15.29	120.82 ± 16.07	125.62 ± 16.64	<0.001
DBP (mmHg, mean ± SD)	69.98 ± 9.53	72.45 ± 10.09	75.10 ± 10.51	78.47 ± 10.92	<0.001
FPG (mg/dL, mean ± SD)	83.49 ± 9.29	86.88 ± 9.40	89.61 ± 9.75	93.58 ± 10.48	＜0. 001
TC (mg/dL, mean ± SD)	165.41 ± 28.64	175.71 ± 30.75	185.95 ± 32.89	198.45 ± 34.94	<0.001
TG (mg/dL, mean ± SD)	51.00 ± 11.26	78.87 ± 12.03	113.51 ± 18.14	199.82 ± 58.97	<0.001
LDL-C (mg/dL, mean ± SD)	50.27 ± 50.02	57.99 ± 54.48	65.55 ± 58.26	71.42 ± 60.79	<0.001
HDL-C (mg/dL, mean ± SD)	53.17 ± 11.91	53.09 ± 11.82	53.08 ± 11.90	53.07 ± 11.79	0.769
ALT (IU/L, median (Q1-Q3)	13.90 (10.90-18.60)	16.00 (12.00-22.80)	19.05 (14.00-28.00)	25.40 (18.00-38.30)	<0.001
AST (IU/L, mean ± SD)	21.74 ± 6.05	22.25 ± 6.41	22.95 ± 6.75	24.25 ± 8.22	<0.001
Scr (umol/L, mean ± SD)	64.70 ± 13.37	68.27 ± 14.45	71.31 ± 14.57	74.29 ± 14.19	<0.001
Smoker					<0.001
Now	1011 (2.05)	2096 (4.13)	3140 (6.30)	4824 (9.41)	
Once	304 (0.62)	536 (1.06)	732 (1.47)	847 (1.65)	
Never	10,591 (21.43)	11,372 (22.40)	10,978 (22.02)	10,556 (20.59)	
Not recorded	37,507 (75.91)	36,762 (72.41)	35,002 (70.21)	35,040 (68.35)	
Drinker					<0.001
Now	108 (0.22)	225 (0.44)	329 (0.66)	581 (1.13)	
Once	1180 (2.39)	1831 (3.61)	2357 (4.73)	3128 (6.10)	
Never	10,618 (21.49)	11,948 (23.54)	12,164 (24.40)	12,518 (24.42)	
Not recorded	37,507 (75.91)	36,762 (72.41)	35,002 (70.21)	35,040 (68.35)	
Family history of diabetes, n (%)					<0.001
No	48,526 (98.20)	49,708 (97.92)	48,804 (97.90)	50,158 (97.84)	
Yes	887 (1.80)	1058 (2.08)	1048 (2.10)	1109 (2.16)	

Values are presented as mean ± SD, median (Q1–Q3) or n (%).

BMI Body-mass index, *SBP* Systolic blood pressure, *DBP* Diastolic blood pressure, *FPG* Fasting plasma glucose, *TC* Total cholesterol, *TG* Triglyceride, *LDL-C* Low-density lipoprotein cholesterol, *HDL-C* High-density lipoprotein cholesterol, *ALT* Alanine aminotransferase, *AST* Aspartate transaminase, *Scr* Serum creatinine

Table 2. The results of univariate analysis

	Statistics	HR (95% CI)	P value
Age (y)	42.08 ± 12.67	1.07 (1.06-1.07)	<0.0001
Gender			
Male	109,236 (54.27%)	Ref	
Female	92,062 (45.73%)	0.51 (0.47-0.55)	<0.0001
BMI (kg/m^2)	23.19 ± 3.32	1.24 (1.23-1.25)	<0.0001
SBP (mmHg)	118.88 ± 16.31	1.04 (1.04-1.04)	<0.0001
DBP (mmHg)	74.03 ± 10.76	1.05 (1.04-1.05)	<0.0001
LDL-C (mg/dL)	61.39 ± 56.64	1.01 (1.00-1.02)	<0.0001
HDL-C (mg/dL)	53.10 ± 11.85	1.00 (1.00-1.01)	0.4881
TC (mg/dl)	181.51 ± 34.19	1.01 (1.01-1.01)	<0.0001
TG (mg/dl)	111.41 ± 64.72	1.01 (1.01-1.01)	<0.0001
FPG (mg/dl)	88.43 ± 10.43	1.15 (1.14-1.15)	<0.0001
TyG index	8.35 ± 0.57	5.78 (5.44-6.14)	<0.0001
Scr (umol/L)	69.68 ± 14.60	1.01 (1.01-1.01)	<0.0001
ALT (U/L)	23.52 ± 19.95	1.01 (1.01-1.01)	<0.0001
AST (U/L)	22.81±6.98	1.01 (1.01-1.02)	<0.0001
Smoker			
Now	11,071 (5.50%)	Ref	
Once	2419 (1.20%)	0.75 (0.56-1.00)	0.0506
Never	43,497 (21.61%)	0.46 (0.40-0.53)	<0.0001
Not recorded	144,311 (71.69%)	0.63 (0.56-0.71)	<0.0001
Drinker			
Now	1243 (0.62%)	Ref	
Once	8496 (4.22%)	0.48 (0.33-0.70)	0.0001
Never	47,248 (23.47%)	0.50 (0.35-0.70)	<0.0001
Not recorded	144,311 (71.69%)	0.54 (0.39-0.76)	0.0003
Family history of DM			
No	197,196 (97.96%)	Ref	
Yes	4102 (2.04%)	1.74 (1.47-2.06)	<0.0001

Relationship between TyG index and future diabetes risk

As shown in Table 3, the effect of TyG index on risk of diabetes was assessed by cox proportional hazard model. In crude model, TyG index demonstrated a strongly positive association with future diabetes risk (HR, 5.78; 95% CI, 5.44–6.14). In model I (adjusted for sex, age and BMI), the positive correlation became relatively weaker (HR, 3.31; 95% CI, 3.09–3.55). In model II (further adjusted for TC, LDL-C, AST, ALT, Scr, DBP, SBP, drinking, smoking and family history of diabetes), the correlation did not change significantly compared with the minor adjustment model (HR, 3.34; 95% CI, 3.11–3.60).

To ensure the robustness of the results, TyG index was processed a categorical variable (quartiles) for sensitivity analysis. There was a graded and positive correlation of TyG index with future risk of diabetes. Compared with the lowest quartile (Q1), increasing TyG index (Q2, Q3, and Q4) was related to increased HR estimates of incident diabetes [HR (95% CI), 1.83 (1.49–2.26); 3.29 (2.70–4.01), and 6.26 (5.15–7.6), respectively].

Nonlinear relationship

As shown in Figure 2, after adjusting for sex, age, BMI, LDL-C, TC, ALT, AST, Scr, SBP, DBP, smoking, drinking, and family history of diabetes, a significant nonlinear relationship was found between TyG index and risk of future diabetes ($p<0.001$), and the slope of the curve showed tendency to ascend with increase of TyG index.

Table 3. Relationship between TyG index and risk of diabetes

Outcomes	Crude model		Model I		Model II	
	HR (95% CI)	P	HR (95% CI)	P	HR (95% CI)	P
TyG index	5.78 (5.44-6.14)	<0.0001	3.31 (3.09-3.55)	<0.0001	3.34 (3.11-3.60)	<0.0001
TyG (quartile)						
Q 1	Ref		Ref		Ref	
Q 2	2.88 (2.34-3.53)	<0.0001	1.84 (1.50-2.27)	<0.0001	1.83 (1.49-2.26)	0.0293
Q 3	7.45 (6.17-9.00)	<0.0001	3.29 (2.70-3.99)	<0.0001	3.29 (2.70-4.01)	0.0004
Q 4	19.94 (16.64-23.88)	<0.0001	6.36 (5.26-7.70)	<0.0001	6.26 (5.15-7.60)	<0.0001
P for trend	<0.0001		<0.0001		<0.0001	

Crude model: adjusted for none.

Model I: adjusted for age, sex and BMI

Model II: adjusted for age, sex, BMI, LDL-C, TC, Scr, AST, ALT, SBP, DBP, drinking, smoking and family history of diabetes

Subgroup analysis

To further investigate the impact of other risk factors on the correlation of TyG index with future diabetes risk, subgroup analyses were carried on according to the following stratification variables: sex, age, BMI, DBP, SBP, smoking, drinking and family history of diabetes. The results of subgroup analyses and interactions were summarized in Table 4. The additive interactions between TyG index and diabetes risk were observed in sex, age, BMI, and SBP (p-value for interaction < 0.05). Stronger correlations were found in participants with age < 40 years, BMI ≥ 18.5 kg/m^2 and < 24 kg/m^2, or SBP < 140 mmHg, or in females. However, significant interactions were not found in DBP, smoking, drinking, or family history of diabetes.

Discussion

This retrospective cohort study revealed that raised TyG index was independently correlated with greater risk of developing diabetes among apparently healthy adults

Table 4. Effect of magnitude of TyG index on diabetes risk stratified by subgroups.

Characteristics	No. of participants	HR (95%CI)	P -value	P for interaction
Age (year)				<0.0001
<40	106,447	4.53 (3.76-5.45)	<0.0001	
>=40, <60	71,176	3.54 (3.19-3.93)	<0.0001	
>=60	23,675	2.67 (2.37-3.00)	<0.0001	
Sex				0.0150
Male	109,236	3.16 (2.90-3.45)	<0.0001	
Female	92,062	3.84 (3.37-4.37)	<0.0001	
BMI(kg/m2)				<0.0001
<18.5	11,593	3.64 (1.53-8.64)	0.0034	
>=18.5, <24	112,241	4.13 (3.62-4.71)	<0.0001	
>=24, <28	60,886	3.22 (2.90-3.58)	<0.0001	
>=28	16,578	3.07 (2.64-3.56)	<0.0001	
SBP(mmHg)				<0.0001
<140	181,383	3.48 (3.19-3.79)	<0.0001	
>=140	19,915	2.89 (2.53-3.29)	<0.0001	
DBP(mmHg)				0.9984
<90	185,636	3.29 (3.04-3.56)	<0.0001	
>=90	15,661	3.43 (2.90-4.05)	<0.0001	
Smoker				0.6979
Now	11,071	3.03 (2.36-3.90)	<0.0001	
Once	2419	4.27 (2.30-7.91)	<0.0001	
Never	43,497	3.40 (2.84-4.08)	<0.0001	
Not recorded	144,311	3.39 (3.13-3.69)	<0.0001	
Drinker				0.2174
Now	1243	5.31 (2.34-12.05)	<0.0001	
Once	8496	3.65 (2.52-5.28)	<0.0001	
Never	47,248	3.33 (2.84-3.90)	<0.0001	
Not recorded	144,311	3.39 (3.13-3.69)	<0.0001	
Family history of diabetes				0.1175
No	197,196	3.39 (3.15-3.65)		
Yes	4102	3.07 (2.10-4.50)		

Note 1: the model was adjusted for sex, age, BMI, LDL-C, TC, Scr, ALT, AST, SBP, DBP, drinking, smoking and family history of diabetes.

Note 2: the model was adjusted for all above variables except the corresponding stratification variable.

healthy adults in China (HR, 3.34; 95% CI, 3.11–3.60). Besides, a significant nonlinear relationship was observed and showed the risk of diabetes tend to ascend with increase of TyG index. Compared with the lowest quartile, individuals with the top quartile of TyG index demonstrated a sixfold greater risk of develop diabetes (Q4 vs. Q1; adjusted HR 6.26, 95% CI 5.15–7.60). Additionally, the results of subgroup analysis revealed this correlation existed regardless of participants being male or female, younger or older, or obese or nonobese, suggesting our results were robust and the TyG index was suitable for a wide range of subjects. Moreover, stronger associations were observed in participants with age < 40 years, BMI ≥ 18.5 kg/m^2 and < 24 kg/m^2, or SBP < 140 mmHg, or in females.

The TyG index, derived from FPG and TG, was proven as a marker of IR in many epidemiological studies [9-13, 21]. Compared with HIEC, the TyG index had high sensitivity (96.5%) as well as good specificity (85.0%) for diagnosing IR in a Mexican population [10], and was a more accurate predictor than HOMA-IR in a Brazilian study[11]. Moreover, consistent with our results, several studies suggested that high TyG index was relevant to future risk of T2DM in different races, as shown in reports from Korea, Singapore, and Europe [15-17, 22]. Similar results were observed in another Chinese cohort study [14] and the trend of nonlinear relationship of TyG index with diabetes risk was generally consistent with our study. However, the study only included 5,706 subjects with normal BMI and was conducted in rural areas. Therefore, its generalizability is relatively limited. This study was based on a large

cohort of 201,298 apparently healthy adults across 32 sites in 11 cities, and is clearly applicable to a relatively wide range of individuals, and provides a stronger basis for clinical promotion and application. Similarly, the risk of diabetes in a Singaporean population elevated progressively across TyG index quartiles (Q) from Q1 to Q4 (Q4 vs. Q1; adjusted HR 5.30, 95% CI 2.21–12.71) [17]. However, potential confounders, such as serum lipid index (LDL-C and TC), drinking, smoking and family history of diabetes, were not sufficiently adjusted, and were notablely relevant to high risk of diabetes [23-28]. Fortunately, these confounding factors were taken into consideration in our study to avoid potential effects on the results.

Subgroup analysis and exploration of interactions is critical for clinical research, to better understand the actual relationships between independent variables and dependent variables [29]. Unfortunately, the related studies described above only used sex, and/or age as stratification factors for subgroup analyses [14-16], and no interactions were observed, which may hinder our understanding of the real association of TyG index with future diabetes risk. In this study, these factors, including BMI, sex, age, DBP, SBP, drinking, smoking and family history of diabetes, were taken as stratified variables, and stronger associations were observed in participants with age < 40 years, BMI ≥ 18.5 kg/m2 and < 24 kg/m2, or SBP < 140 mmHg, or in females. This association was particularly obvious in females, and was consistent with the cohort study by Zhang et al [14]. This may be because serum lipids in female hepatocytes were higher than that in male hepatocytes under fasting and glucose lipid loading [30, 31]. In clinical practice,

obese and older individuals are generally considered the primary targets for diabetes screening. However, based on subgroup analysis, the TyG index appeared to be more sensitive for predicting risk of diabetes in younger individuals and those with normal BMI or SBP, suggesting it may be promising for screening risk of future diabetes, especially in individuals without high-risk factors such as hypertension, obesity and older age.

Islet β-cell dysfunction and IR remain the core pathological trait of T2DM [32]. Interestingly, the TyG index, besides being a substitute of IR, is associated with susceptibility of β-cells to glucotoxicity and lipotoxicity. Evidence suggested that elevated glucose levels can induced reactive oxygen species generation on islet β-cells, which in turn cause oxidative stress and β-cells dysfunction, and then lead to IR and T2DM [33-35]. Other studies revealed that long-term high free fatty acid content was related to prolonged exposure of TG in pancreatic islets, which may impair pancreatic β-cell function [36-38]. Furthermore, glycotoxicity and lipotoxicity were interactive rather than independent adverse effects on pancreatic β-cell [39-41]. Long term exposure of pancreatic beta cells to high fatty acids concentrations could result in impaired glucose-induced insulin secretion [42, 43] and increased β-cell death [44]. An intervention study confirmed that patients with impaired glucose metabolism had improved insulin secretion ability after being treated with n-3 fatty acids [45]. Besides, IR is largely attributable to the impairment of insulin-stimulated glucose absorption into skeletal muscle. When TG levels in peripheral blood and skeletal muscle were

significantly increased, glucose metabolism in skeletal muscle would be impaired [46]. Therefore, to a certain extent, the TyG index reflects muscle IR [47].

This study had several advantages. First, it was based on a large sample cohort study with broad age spectrum. Therefore, there were sufficient subjects for analysis to guarantee dependability and robustness of results. Furthermore, the results are applicable to a relatively wide range of individuals. Other similar cohort studies had relatively small sample sizes and populations that tended to be older. Second, taking TyG index as continuous variable and categorical variable respectively, sensitivity analysis and trend test were carried out to improve the reliability of results and avoid the contingency in data analysis. Finally, subgroup analyse and interaction test were conducted to further prove the dependability of the results and identify potential interactions with other variables. Interestingly, we observed that the TyG index appears to be more sensitive for predicting risk of diabetes in females, younger individuals, and those with normal BMI or SBP. Therefore, the TyG index may represent a reliable predictor for screening diabetes in individuals without high-risk factors such as older age, obesity, and hypertension. More evidence is needed to further validate our results.

This study also had limitations. Firstly, diabetes was diagnosed depending on FPG ≥ 7.0 mmol / L or self-reported diabetes, rather than by glycosylated hemoglobin or 2-hour oral glucose tolerance test, which was probably underestimated. Secondly, this study did not distinguish between types of diabetes. However, these findings may be more applicable to T2DM, which accounts for approximately 90-95% of all diabetes

cases. Thirdly, as this large cohort study was conducted in China, our findings can not be generalized to other races and certain populations, such as children and pregnant women. Finally, the present report was a secondary analysis on the basis of existing database, and although numerous confounding factors had been adjusted, some variables not included in the database, such as physical activity, dietary factors, and lipid-lowering agents, failed to be adjusted. Therefore, potential effects of these residual confounding factors on the results cannot be ignored.

Conclusions

This study manifested that elevated TyG index was independently correlated with increased risk of developing diabetes in Chinese adults. Besides, these findings expand our current knowledge that the TyG index seems to be more sensitive for predicting risk of diabetes in women, younger individuals, and those with normal BMI or SBP. The TyG index may therefore represent a reliable predictor for screening individuals at early diabetes risk, especially in people without high-risk factors such as older age, hypertension and obesity.

Figure Legends

Figure 1. Kaplan-Meier analysis of future diabetes risk according to TyG index quartiles (log-rank, $p < 0.0001$).

Figure 2. A nonlinear relationship of TyG index with risk of future diabetes. Note: the model has been adjusted for sex, age, BMI, AST, ALT, LDL-C, TC, Scr, SBP, DBP, drinking, smoking and family history of diabetes.

Abbreviations

TyG index triglyceride-glucose index, *BMI* body mass index, *FPG* fasting plasma glucose,*TG* triglyceride, *TC* total cholesterol, *HDL-C* high density lipoprotein cholesterol, *LDL-C* low density lipoprotein cholesterol, *Scr* serum creatinine, *ALT* alanine aminotransferase, *AST* aspartate aminotransferase, *DBP* diastolic blood pressure, *SBP* systolic blood pressure, *IR* insulin resistance

Ethics approval and consent to participate

In the original article [17], Chen, et, al. have declared the study was authorized by the Rich Healthcare Group Review Board and complied with the declaration of Helsinki. Considering that the study was retrospective, no participants were required to participate in any part of the study.

Consent for publication

Not applicable.

Availability of data and materials

Data can be downloaded from 'DATADRYAD' database (www.Datadryad.org).

Competing interests

None

Funding

This study was supported by Natural Science Foundation of Hebei Province (No. H2019307108).

Authors' contributions

XLL and HJM designed this study. XLL, GLL and TTC conducted data cleaning and statistical analysis. XLL and JL made the result interpretation. XLL, GYS and HJM participated in the discussion; XLL drafted the manuscript, and HJM revised this manuscript. All authors read and approved the final manuscript.

Acknowledgements

Not applicable.

References

1. Yoon KH, Lee JH, Kim JW, et al. Epidemic obesity and type 2 diabetes in Asia. Lancet. 2006;368(9548):1681-1688. https://doi.org/10.1016/S0140-6736(06)69703-1.

2. Global Burden of Metabolic Risk Factors for Chronic Diseases Collaboration. Cardiovascular disease, chronic kidney disease, and diabetes mortality burden of cardiometabolic risk factors from 1980 to 2010: a comparative risk assessment. Lancet Diabetes Endocrinol. 2014;2(8):634-647. https://doi.org/10.1016/S 2213-8587 (14)70102-0.

3. Seuring T, Archangelidi O, Suhrcke M. The Economic Costs of Type 2 Diabetes: A Global Systematic Review. Pharmacoeconomics. 2015;33(8):811-831. https://doi. org/10.1007/s40273-015-0268-9.

4. Shaw JE, Sicree RA, Zimmet PZ. Global estimates of the prevalence of diabetes for 2010 and 2030. Diabetes Res Clin Pract. 2010;87(1):4-14. https://doi.org/ 10. 1016/j.diabres.2009.10.007

5. Warram JH, Martin BC, Krolewski AS, Soeldner JS, Kahn CR. Slow glucose

removal rate and hyperinsulinemia precede the development of type II diabetes in the offspring of diabetic parents. Ann Intern Med. 1990;113(12):909-915. https://doi.org/10.7326/0003-4819-113-12-909

6. Defronzo RA. Banting Lecture. From the triumvirate to the ominous octet: a new paradigm for the treatment of type 2 diabetes mellitus. Diabetes. 2009;58(4):773-795. https://doi.org/10.2337/db09-9028

7. Scheen AJ. Pathophysiology of type 2 diabetes. Acta Clin Belg. 2003;58(6):335-341. https://doi.org/10.1179/acb.2003.58.6.001

8. DeFronzo RA, Tobin JD, Andres R. Glucose clamp technique: a method for quantifying insulin secretion and resistance. Am J Physiol. 1979;237(3):E214-E223. https://doi.org/10.1152/ajpendo.1979.237.3.E214

9. Simental-Mendía LE, Rodríguez-Morán M, Guerrero-Romero F. The product of fasting glucose and triglycerides as surrogate for identifying insulin resistance in apparently healthy subjects. Metab Syndr Relat Disord. 2008;6(4):299-304. https://doi.org/10.1089/met.2008.0034.

10. Guerrero-Romero F, Simental-Mendía LE, González-Ortiz M, et al. The product of triglycerides and glucose, a simple measure of insulin sensitivity. Comparison with the euglycemic-hyperinsulinemic clamp. J Clin Endocrinol Metab. 2010;95(7):3347-3351. https://doi.org/10.1210/jc.2010-0288

11. Vasques AC, Novaes FS, de Oliveira Mda S, et al. TyG index performs better than HOMA in a Brazilian population: a hyperglycemic clamp validated study. Diabetes Res

Clin Pract. 2011;93(3):e98-e100. https://doi.org/10.1016/j.diabres. 2011.05.030.

12. Guerrero-Romero F, Villalobos-Molina R, Jiménez-Flores JR, et al. Fasting Triglycerides and Glucose Index as a Diagnostic Test for Insulin Resistance in Young Adults. Arch Med Res. 2016;47(5):382-387. https://doi.org/10.1016/j.arcmed.2016.08.012

13. Mazidi M, Kengne AP, Katsiki N, Mikhailidis DP, Banach M. Lipid accumulation product and triglycerides/glucose index are useful predictors of insulin resistance. J Diabetes Complications. 2018;32(3):266-270. https://doi.org/10.1016/j.jdiacomp.2017.10.007

14. Zhang M, Wang B, Liu Y, et al. Cumulative increased risk of incident type 2 diabetes mellitus with increasing triglyceride glucose index in normal-weight people: The Rural Chinese Cohort Study. Cardiovasc Diabetol. 2017;16(1):30. https://doi.org/10.1186/s12933-017-0514-x

15. Lee SH, Kwon HS, Park YM, et al. Predicting the development of diabetes using the product of triglycerides and glucose: the Chungju Metabolic Disease Cohort (CMC) study. PLoS One. 2014;9(2):e90430. Published 2014 Feb 28. https://doi.org/10.1371/journal.pone.0090430

16. Lee JW, Lim NK, Park HY. The product of fasting plasma glucose and triglycerides improves risk prediction of type 2 diabetes in middle-aged Koreans. BMC Endocr Disord. 2018;18(1):33. https://doi.org/10.1186/s12902-018-0259-x

17. Low S, Khoo KCJ, Irwan B, et al. The role of triglyceride glucose index in development of Type 2 diabetes mellitus. Diabetes Res Clin Pract. 2018;143:43-49. https://doi.org/10.1016/j.diabres.2018.06.006

18. Chen Y, Zhang XP, Yuan J, et al. Association of body mass index and age with incident diabetes in Chinese adults: a population-based cohort study. BMJ Open. 2018;8(9):e021768. https://doi.org/10.1136/bmjopen- 2018-021768.

19. Zhang N, Hu X, Zhang Q, et al. Non-high-density lipoprotein cholesterol: High-density lipoprotein cholesterol ratio is an independent risk factor for diabetes mellitus: Results from a population-based cohort study. J Diabetes. 2018;10(9): 708-714. https://doi.org/10.1111/1753-0407.12650

20. Erviti J, Alonso A, Oliva B, et al. Oral bisphosphonates are associated with increased risk of subtrochanteric and diaphyseal fractures in elderly women: a nested case-control study. BMJ Open. 2013;3(1):e002091. https://doi.org/10.1136//bmjopen-2012-002091

21. Sánchez-García A, Rodríguez-Gutiérrez R, Mancillas-Adame L, et al. Diagnostic Accuracy of the Triglyceride and Glucose Index for Insulin Resistance: A Systematic Review. Int J Endocrinol. 2020;2020:4678526. https://doi.org/10.1155/2020/4678526

22. Navarro-González D, Sánchez-Íñigo L, Pastrana-Delgado J, Fernández-Montero A, Martinez JA. Triglyceride-glucose index (TyG index) in comparison with fasting plasma glucose improved diabetes prediction in patients with normal fasting glucose: The Vascular-Metabolic CUN cohort. Prev Med. 2016;86:99-105.

https://doi.org/10.1016/j.ypmed.2016.01.022

23. Athyros VG, Doumas M, Imprialos KP, et al. Diabetes and lipid metabolism. Hormones (Athens). 2018;17(1):61-67. https://doi.org/10.1007/s42000-018-0014-8

24. Tangvarasittichai S. Oxidative stress, insulin resistance, dyslipidemia and type 2 diabetes mellitus. World J Diabetes. 2015;6(3):456-480. https://doi.org/10.4239/wjd.v6.i3.456

25. Namayandeh SM, Karimi A, Fallahzadeh H, et al. The incidence rate of diabetes mellitus (type II) and its related risk factors: A 10-year longitudinal study of Yazd Healthy Heart Cohort (YHHC), Iran. Diabetes Metab Syndr. 2019;13(2):1437-1441. https://doi.org/10.1016/j.dsx.2019.02.012

26. Radzeviciene L, Ostrauskas R. Smoking habits and the risk of type 2 diabetes: a case-control study. Diabetes Metab. 2009;35(3):192-197. https://doi.org/10.1016/j.diabet.2008.11.001

27. Brath H, Kaser S, Tatschl C, et al. Smoking, alcohol and diabetes (Update 2019). Wien Klin Wochenschr. 2019;131(Suppl 1):67-70. https://doi.org/10.1007/s00508 -019-1455-z

28. Zhang Y, Chen H, Lu H, et al. Prevalence and risk of diabetes based on family history in the Shanghai High-Risk Diabetic Screen (SHiDS) study. Diabet Med. 2016;33(12):1705-1711. https://doi.org/10.1111/dme.13013

29. Vandenbroucke JP, von Elm E, Altman DG, et al. Strengthening the Reporting of Observational Studies in Epidemiology (STROBE): explanation and elaboration. Ann

Intern Med. 2007;147(8):W163-W194. https://doi.org/10.7326/ 0003-4819-147-8-200710160-00010-w1

30. Greenman Y, Golani N, Gilad S, Yaron M, Limor R, Stern N. Ghrelin secretion is modulated in a nutrient- and gender-specific manner. Clin Endocrinol (Oxf). 2004;60(3):382-388. https://doi.org/10.1111/j.1365-2265.2004.01993.x

31. Machann J, Thamer C, Schnoedt B, et al. Age and gender related effects on adipose tissue compartments of subjects with increased risk for type 2 diabetes: a whole body MRI/MRS study. MAGMA. 2005;18(3):128-137. https://doi.org/10.1007/s10334-005-0104-x

32. Sánchez-García A, Rodríguez-Gutiérrez R, Mancillas-Adame L, et al. Diagnostic Natural history of β-cell adaptation and failure in type 2 diabetes. Mol Aspects Med. 2015;42:19-41. https://doi.org/10.1016/j.mam.2014.12.002

33. Robertson RP, Harmon J, Tran PO, Poitout V. Beta-cell glucose toxicity, lipotoxicity, and chronic oxidative stress in type 2 diabetes. Diabetes. 2004;53 Suppl 1:S119-S124. https://doi.org/10.2337/diabetes.53.2007.s119

34. Robertson RP, Harmon J, Tran PO, Tanaka Y, Takahashi H. Glucose toxicity in beta-cells: type 2 diabetes, good radicals gone bad, and the glutathione connection. Diabetes. 2003;52(3):581-587. https://doi.org/10.2337/diabetes.52.3.581

35. Robertson R, Zhou H, Zhang T, Harmon JS. Chronic oxidative stress as a mechanism for glucose toxicity of the beta cell in type 2 diabetes. Cell Biochem Biophys. 2007;48(2-3):139-146. https://doi.org/10.1007/s12013-007-0026-5

36. Mason TM, Goh T, Tchipashvili V, et al. Prolonged elevation of plasma free fatty acids desensitizes the insulin secretory response to glucose in vivo in rats. Diabetes. 1999;48(3):524-530. https://doi.org/10.2337/diabetes.48.3.524

37. Jacqueminet S, Briaud I, Rouault C, Reach G, Poitout V. Inhibition of insulin gene expression by long-term exposure of pancreatic beta cells to palmitate is dependent on the presence of a stimulatory glucose concentration. Metabolism. 2000;49(4):532-536. https://doi.org/10.1016/s0026-0495(00)80021-9

38. Maedler K, Spinas GA, Dyntar D, Moritz W, Kaiser N, Donath MY. Distinct effects of saturated and monounsaturated fatty acids on beta-cell turnover and function. Diabetes. 2001;50(1):69-76. https://doi.org/10.2337/diabetes.50.1.69

39. Weir GC. Glucolipotoxicity, β-Cells, and Diabetes: The Emperor Has No Clothes. Diabetes. 2020;69(3):273-278. https://doi.org/10.2337/db19-0138

40. Lytrivi M, Castell AL, Poitout V, Cnop M. Recent Insights Into Mechanisms of β-Cell Lipo- and Glucolipotoxicity in Type 2 Diabetes. J Mol Biol. 2020;432(5):1514-1534. https://doi.org/10.1016/j.jmb.2019.09.016

41. Fu J, Cui Q, Yang B, et al. The impairment of glucose-stimulated insulin secretion in pancreatic β-cells caused by prolonged glucotoxicity and lipotoxicity is associated with elevated adaptive antioxidant response. Food Chem Toxicol. 2017;100:161-167. https://doi.org/10.1016/j.fct.2016.12.016

42. Zhou YP, Grill VE. Long-term exposure of rat pancreatic islets to fatty acids inhibits glucose-induced insulin secretion and biosynthesis through a glucose fatty acid

cycle. J Clin Invest. 1994;93(2):870-876. https://doi.org/10.1172/JCI117042

43. Zhou YP, Grill V. Long term exposure to fatty acids and ketones inhibits B-cell functions in human pancreatic islets of Langerhans. J Clin Endocrinol Metab. 1995;80(5):1584-1590. https://doi.org/10.1210/jcem.80.5.7745004

44. Lupi R, Dotta F, Marselli L, et al. Prolonged exposure to free fatty acids has cytostatic and pro-apoptotic effects on human pancreatic islets: evidence that beta-cell death is caspase mediated, partially dependent on ceramide pathway, and Bcl-2 regulated. Diabetes. 2002;51(5):1437-1442. https://doi.org/10.2337/diabetes.51.5.1437

45. Sawada T, Tsubata H, Hashimoto N, et al. Effects of 6-month eicosapentaenoic acid treatment on postprandial hyperglycemia, hyperlipidemia, insulin secretion ability, and concomitant endothelial dysfunction among newly-diagnosed impaired glucose metabolism patients with coronary artery disease. An open label, single blinded, prospective randomized controlled trial. Cardiovasc Diabetol. 2016;15(1):121. https://doi.org/10.1186/s12933-016-0437-y

46. Kelley DE, Goodpaster BH, Storlien L. Muscle triglyceride and insulin resistance. Annu Rev Nutr. 2002;22:325-346. https://doi.org/10.1146/annurev.nutr.22.010402.102912

47. Tripathy D, Almgren P, Tuomi T, Groop L. Contribution of insulin-stimulated glucose uptake and basal hepatic insulin sensitivity to surrogate measures of insulin sensitivity. Diabetes Care. 2004;27(9):2204-2210. https://doi.org/10.2337/diacare.27.9.2204.

Association of triglyceride-glucose index and the risk of incident diabetes: a secondary analysis based on a Chinese cohort study

ORIGINALITY REPORT

19%

SIMILARITY INDEX

PRIMARY SOURCES

1	www.researchsquare.com<br>Internet	299 words — 5%
2	www.hindawi.com<br>Internet	58 words — 1%
3	lipidworld.biomedcentral.com<br>Internet	48 words — 1%
4	link.springer.com<br>Internet	46 words — 1%
5	datadryad.org<br>Internet	33 words — 1%
6	"56th EASD Annual Meeting of the European Association for the Study of Diabetes", Diabetologia, 2020<br>Crossref	33 words — 1%
7	cardiab.biomedcentral.com<br>Internet	20 words — < 1%
8	Serena Low, Kay Chin Jonathon Khoo, Bastari Irwan, Chee Fang Sum, Tavintharan Subramaniam, Su Chi Lim, Tack Keong Michael Wong. "The Role of Triglyceride Glucose Index in Development of Type 2 Diabetes Mellitus", Diabetes Research and Clinical Practice, 2018	18 words — < 1%

Crossref

9 Wenrui Shi, Liying Xing, Li Jing, Yuanmeng Tian, Han Yan, Qun Sun, Dong Dai, Lei Shi, Shuang Liu. "Value of triglyceride-glucose index for the estimation of ischemic stroke risk: Insights from a general population", Nutrition, Metabolism and Cardiovascular Diseases, 2020 — 17 words — < 1%
Crossref

10 www.mdpi.com — 16 words — < 1%
Internet

11 Kunyan Wang, Gui He, Yuehua Zhang, Jingjing Yin, Yali Yan, Ye Zhang, Kaijuan Wang. "Association of triglyceride-glucose index and its interaction with obesity on hypertension risk in Chinese: a population-based study", Journal of Human Hypertension, 2020 — 15 words — < 1%
Crossref

12 Song Jian, Nie Su-Mei, Chen Xue, Zhang Jie, Wu Xue-sen. "Association and interaction between triglyceride–glucose index and obesity on risk of hypertension in middle-aged and elderly adults", Clinical and Experimental Hypertension, 2017 — 14 words — < 1%
Crossref

13 Xiuping Xuan, Masahide Hamaguchi, Qiuli Cao, Okamura Takuro et al. "U-Shaped Association Between the Triglycerides-Glucose index and the Risk of Incident Diabetes in Apparently Healthy Population: A Population-based Cohort Study", Research Square, 2020 — 14 words — < 1%
Crossref

14 Ho Jin Kim, Jun Sung Moon, Il Rae Park, Joong Hee Kim, Ji Sung Yoon, Kyu Chang Won, Hyoung Woo Lee. "A Novel Index Using Soluble CD36 Is Associated with the Prevalence of Type 2 Diabetes Mellitus: Comparison Study with Triglyceride-Glucose Index", Endocrinology and Metabolism, 2017 — 14 words — < 1%
Crossref

15 pubmed.ncbi.nlm.nih.gov — 14 words — < 1%
Internet

16 www.future-science.com
Internet
13 words — < 1%

17 Zeyin Lin, Dongming Guo, Juntian Chen, Baoqun Zheng. "A nomogram for predicting 5-year incidence of type 2 diabetes in a Chinese population", Endocrine, 2019
Crossref
12 words — < 1%

18 academic.oup.com
Internet
12 words — < 1%

19 Alejandro Fernandez-Montero, David García-Ros, Ana Sánchez-Tainta, Ana Rodriguez-Mourille, Antonio Vela, Stefanos N. Kales. "Burnout Syndrome and Increased Insulin Resistance", Journal of Occupational and Environmental Medicine, 2019
Crossref
12 words — < 1%

20 clinicaltrials.gov
Internet
12 words — < 1%

21 www.ncbi.nlm.nih.gov
Internet
12 words — < 1%

22 "Abstracts of the 47th Annual Meeting of the EASD, Lisbon 2011", Diabetologia, 2011
Crossref
12 words — < 1%

23 Wenrui Shi, Liying Xing, Li Jing, Yuanmeng Tian, Shuang Liu. "Usefulness of Triglyceride-glucose Index for estimating Hyperuricemia risk: Insights from a general Population", Postgraduate Medicine, 2019
Crossref
12 words — < 1%

24 www.poitoutlab.ca
Internet
11 words — < 1%

25 Chi Chen, Jia-Lin Dai. "Triglyceride to high-density lipoprotein cholesterol (HDL-C) ratio and arterial stiffness in Japanese population: a secondary analysis based on a cross-sectional study", Lipids in Health and Disease, 2018
Crossref
11 words — < 1%

26	Vik, A.. "Serum osteoprotegerin is inversely associated with carotid plaque echogenicity in humans", Atherosclerosis, 200703<br>Crossref	11 words — < 1%
27	core.ac.uk<br>Internet	10 words — < 1%
28	onlinelibrary.wiley.com<br>Internet	10 words — < 1%
29	www.bioline.org.br<br>Internet	10 words — < 1%
30	Yang Zou, Meng Yu, Guotai Sheng. "Association between fasting plasma glucose and nonalcoholic fatty liver disease in a nonobese Chinese population with normal blood lipid levels: a prospective cohort study", Lipids in Health and Disease, 2020<br>Crossref	9 words — < 1%
31	smj.psmmc.med.sa<br>Internet	9 words — < 1%
32	Ying Chen, Xiao-Ping Zhang, Jie Yuan, Bo Cai et al. "Association of body mass index and age with incident diabetes in Chinese adults: a population-based cohort study", BMJ Open, 2018<br>Crossref	9 words — < 1%
33	Enqian Liu, Yaping Weng, Aiming Zhou, Chunlai Zeng. "The triglyceride-glucose index (TyG) and Nonalcoholic fatty liver in the Japanese population: a retrospective cross-sectional study", Research Square, 2020<br>Crossref	9 words — < 1%
34	www.nice.org.uk<br>Internet	9 words — < 1%
	"Supplement 2 September 2011", Intensive Care Medicine, 2011	

35 Crossref

9 words — < 1%

36 Sangsang Li, Bingxin Guo, Huanan Chen, Zhan Shi, Yapeng Li, Qingfeng Tian, Songhe Shi. "The role of the triglyceride (triacylglycerol) glucose index in the development of cardiovascular events: a retrospective cohort analysis", Scientific Reports, 2019
Crossref

9 words — < 1%

37 my.amcp.org
Internet

9 words — < 1%

38 bmcpublichealth.biomedcentral.com
Internet

8 words — < 1%

39 Ren, Yongcheng, Ming Zhang, Jingzhi Zhao, Chongjian Wang, Xinping Luo, Jiatong Zhang, Tian Zhu, Xi Li, Lei Yin, Chao Pang, Tianping Feng, Bingyuan Wang, Lu Zhang, Linlin Li, Xiangyu Yang, Hongyan Zhang, and Dongsheng Hu. "Association of the hypertriglyceridemic-waist phenotype and type 2 diabetes mellitus among adults in China", Journal of Diabetes Investigation, 2016.
Crossref

8 words — < 1%

40 www.medsci.org
Internet

8 words — < 1%

41 worldwidescience.org
Internet

8 words — < 1%

42 tessera.spandidos-publications.com
Internet

8 words — < 1%

43 www.nhlbi.nih.gov
Internet

8 words — < 1%

44 Panya Chamroonkiadtikun, Thareerat Ananchaisarp, Worawit Wanichanon. "The triglyceride-glucose index, a predictor of type 2 diabetes development: A retrospective

8 words — < 1%

cohort study", Primary Care Diabetes, 2020
Crossref

45 Weihang Lu, Wei Guo, Jie Liu, Yangyang Ge, Dan Rong, Bai He, Jianfei Dong, Xin Jia. "Consistency of Proximal-to-Distal Tapering of Descending Thoracic Aortic Diameter: Quantification Using a Novel Computer Tomography Based Assessment", Annals of Vascular Surgery, 2019
Crossref
8 words — < 1%

46 bmcinfectdis.biomedcentral.com
Internet
8 words — < 1%

47 Susalit, E.. "Olive (Olea europaea) leaf extract effective in patients with stage-1 hypertension: Comparison with Captopril", Phytomedicine, 20110215
Crossref
8 words — < 1%

48 epdf.tips
Internet
8 words — < 1%

49 www.oncotarget.com
Internet
8 words — < 1%

50 Mirella Hietaniemi, Seppo M. Pöykkö, Olavi Ukkola, Markku Päivänsalo, Y. Antero Kesäniemi. "IGF-I concentrations are positively associated with carotid artery atherosclerosis in women", Annals of Medicine, 2009
Crossref
8 words — < 1%

51 Qi Zhao, Ting-Yu Zhang, Yu-Jing Cheng, Yue Ma, Ying-Kai Xu, Jia-Qi Yang, Yu-jie Zhou. "Impacts of triglycerides-glucose index on prognosis of patients with type 2 diabetes mellitus and non-ST-segment elevation acute coronary syndrome: results from an observational cohort study in China", Research Square, 2020
Crossref
7 words — < 1%

52 Zhuangsen Chen, Haofei Hu, Miaoling Chen, Xueying Luo, Weili Yao, Qian Liang, Fan Yang, Xinyu Wang. "Association of Triglyceride to high-density lipoprotein cholesterol ratio and incident of diabetes mellitus: a secondary
7 words — < 1%

retrospective analysis based on a Chinese cohort study", Lipids in Health and Disease, 2020
Crossref

---

53 Xiaoteng Ma, Lisha Dong, Qiaoyu Shao, Yujing Cheng, Sai Lv, Yan Sun, Hua Shen, Zhijian Wang, Yujie Zhou, Xiaoli Liu. "Triglyceride glucose index for predicting cardiovascular outcomes after percutaneous coronary intervention in patients with type 2 diabetes mellitus and acute coronary syndrome", Cardiovascular Diabetology, 2020 — 7 words — < 1%
Crossref

---

54 S. K. Park, S. D. Harlow, H. Zheng, C. Karvonen-Gutierrez, R. C. Thurston, K. Ruppert, I. Janssen, J. F. Randolph. "Association between changes in oestradiol and follicle-stimulating hormone levels during the menopausal transition and risk of diabetes", Diabetic Medicine, 2017 — 7 words — < 1%
Crossref

---

55 Wenrui Shi, Shuang Liu, Li Jing, Yuanmeng Tian, Liying Xing. "Estimate of reduced glomerular filtration rate by triglyceride-glucose index: insights from a general Chinese population", Postgraduate Medicine, 2019 — 7 words — < 1%
Crossref

---

56 Yang Wu, Haofei Hu, Jinlin Cai, Runtian Chen, Xin Zuo, Heng Cheng, Yongcheng He, Dewen Yan. "Association of hypertension and incident diabetes in Chinese adults: a retrospective cohort study using propensity-score matching", Research Square, 2020 — 6 words — < 1%
Crossref

---

57 Yaqi Yin, Sisi Ye, Haibin Wang, Bing Li, Anping Wang, Wenhua Yan, Jingtao Dou, Yiming Mu. "Red blood cell distribution width and the risk of being in poor glycemic control among patients with established type 2 diabetes", Therapeutics and Clinical Risk Management, 2018 — 6 words — < 1%
Crossref

---

58 Takuro Okamura, Yoshitaka Hashimoto, Masahide Hamaguchi, Akihiro Obora, Takao Kojima, Michiaki Fukui. "Triglyceride–glucose index is a predictor of incident chronic — 6 words — < 1%

kidney disease: a population-based longitudinal study", Clinical and Experimental Nephrology, 2019
Crossref

59 Zhilin Liang, Haofei Hu, Xunxun Wang, Xianxiong Chen, Zhigang Liu, Min Zhang. "Association of Alanine Transaminase to Aspartate Aminotransferase Ratio and Incident Diabetes in Chinese Adults: A Second Retrospective Cohort Study", Research Square, 2020
Crossref

6 words — < 1%

60 Buyuan Dong, Yuqing Mao, Zhengyang Li, Fujun Yu. "The value of atherogenic index of plasma in non-obese patients with non-alcoholic fatty liver disease: a secondary analysis based on a cross-sectional study.", Research Square, 2020
Crossref

6 words — < 1%

EXCLUDE QUOTES	OFF	EXCLUDE MATCHES	OFF
EXCLUDE BIBLIOGRAPHY	ON		
