# Supplementary material for: Association between triglyceride-glucose index and risk of incident diabetes: a secondary analysis based on a Chinese cohort study: TyG index and incident diabetes
Source: Lipids Health Dis. 2020 Nov 8;19:236. doi: 10.1186/s12944-020-01403-7 (PMC7649000; doi:10.1186/s12944-020-01403-7)
Supplement: Supplementary file 2 — Additional file 2. [file 12944_2020_1403_MOESM2_ESM.pdf]

Certificate of Editing

Edited provisional title
Association between triglyceride-glucose index and risk of incident diabetes: a secondary analysis based on a Chinese cohort study

Client name and institution
Huijuan Ma, Department of Internal Medicine, Hebei Medical University, Shijiazhuang 050017, Hebei, China; Hebei Key Laboratory of Metabolic Diseases, Hebei General Hospital, Shijiazhuang 050051, Hebei, China

Date Completed
2020-08-21

Identification code
87222

Certificate issued by
Koji Yamashita
Managing Director and CEO

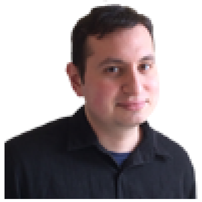

Expert Editor: Richard Robins
2012 PhD Physiology
McGill University
Biochemistry and Cell Biology, Pharmacology and Pharmaceutical Sciences, Cardiorespiratory Medicine and Haematology

www.liwenbianji.cn
While this certificate confirms the authors have used Edanz's editing services, we cannot guarantee that additional changes have not been made after our edits.
