# Supplementary material for: Association between triglyceride-glucose index and risk of incident diabetes: a secondary analysis based on a Chinese cohort study: TyG index and incident diabetes
Source: Lipids Health Dis. 2020 Nov 8;19:236. doi: 10.1186/s12944-020-01403-7 (PMC7649000; doi:10.1186/s12944-020-01403-7)
Supplement: Supplementary file 3 — Additional file 3. [file 12944_2020_1403_MOESM3_ESM.doc]

**Appendix:**

1. The analysis results based on population with BMI < 18.5 kg/m^2^ (Table 1)

2. The analysis results based on population with BMI ≥ 28 kg/m^2^ (Table 2-3).

3. The analysis results based on population with 18.5 < BMI < 40 kg/m^2^ (Table 4-7).

1. The analysis results based on population with BMI < 18.5 kg/m^2^

**Table 1.** Relationship between TyG index and risk of diabetes based on population with BMI <18.5

kg/m^2^ (n=11,593)

| Outcomes | Crude model | |  | Model Ⅰ | |  | Model Ⅱ | |
| --- | --- | --- | --- | --- | --- | --- | --- | --- |
|  | HR (95% CI) | P |  | HR (95% CI) | P |  | HR (95% CI) | P |
| TyG index | 5.95 (2.80, 12.67) | <0.0001 |  | 3.64 (1.53, 8.65) | <0.0001 |  | 3.38 (1.50, 7.67) | <0.0001 |
| TyG (quartile) |  |  |  |  |  |  |  |  |
| Q 1 | Ref |  |  | Ref |  |  | Ref |  |
| Q 2 | 2.71 (0.83, 8.86) | <0.0001 |  | 2.52 (0.75, 8.50) | <0.0001 |  | 2.53 (0.76, 8.44) | <0.0001 |
| Q 3 | 3.33 (1.74, 6.96) | <0.0001 |  | 2.75 (0.42, 8.27) | <0.0001 |  | 2.73 (1.39, 8.23) | <0.0001 |
| Q 4 | 6.23 (2.08, 18.60) | <0.0001 |  | 3.93 (1.18, 13.09) | <0.0001 |  | 3.80 (1.18, 12.28) | <0.0001 |
| P for trend | 0.0019 |  |  | 0.0031 |  |  | 0.0028 |  |

**Crude model:** adjusted for none.

**Model Ⅰ:** adjusted for age, sex and BMI

**Model Ⅱ:** adjusted for age, sex, BMI, LDL-C, TC, Scr, AST, ALT, SBP, DBP, drinking, smoking and family history of diabetes

2. The analysis results based on population with BMI≥28 kg/m^2^ (Table 2-3).

**Table 2.** Relationship between TyG index and risk of diabetes based on population with BMI≥28

**kg/m^2^ (n=16,578)**

| Outcomes | Crude model | |  | Model Ⅰ | |  | Model Ⅱ | |
| --- | --- | --- | --- | --- | --- | --- | --- | --- |
|  | HR (95% CI) | P |  | HR (95% CI) | P |  | HR (95% CI) | P |
| TyG index | 3.15 (2.74, 3.63) | <0.0001 |  | 2.93 (2.54, 3.38) | <0.0001 |  | 3.06 (2.64, 3.56) | <0.0001 |
| TyG (quartile) |  |  |  |  |  |  |  |  |
| Q 1 | Ref |  |  | Ref |  |  | Ref |  |
| Q 2 | 1.92 (1.48, 2.48) | <0.0001 |  | 1.78 (1.38, 2.30) | <0.0001 |  | 1.81 (1.40, 2.34) | <0.0001 |
| Q 3 | 3.02 (2.38, 3.85) | <0.0001 |  | 2.73 (2.14, 3.47) | <0.0001 |  | 2.77 (2.16, 3.54) | <0.0001 |
| Q 4 | 4.65 (3.69, 5.86) | <0.0001 |  | 4.09 (3.24, 5.17) | <0.0001 |  | 4.27 (3.36, 5.43) | <0.0001 |
| P for trend | <0.0001 |  |  | <0.0001 |  |  | <0.0001 |  |

**Crude model:** adjusted for none.

**Model Ⅰ:** adjusted for age, sex and BMI

**Model Ⅱ:** adjusted for age, sex, BMI, LDL-C, TC, Scr, AST, ALT, SBP, DBP, drinking, smoking and family history of diabetes

**Table 3.** Effect of magnitude of TyG index on diabetes risk stratified by BMI (BMI≥28 kg/m^2^,

n=16,578)

| **Outcomes** | **BMI<32 (kg/m^2^)**  **(n=14388)** | |  | **BMI >=32 (kg/m^2^)**  **(n=2190)** | |
| --- | --- | --- | --- | --- | --- |
|  | **HR (95% CI)** | **P** |  | **HR (95% CI)** | **P** |
| TyG index | 3.11 (2.64, 3.68) | <0.0001 |  | 3.36 (2.33, 4.85) | <0.0001 |
| TyG (quartile) |  |  |  |  |  |
| Q 1 | Ref |  |  | Ref |  |
| Q 2 | 1.75 (1.32, 2.33) | <0.0001 |  | 2.48 (1.31, 4.69) | 0.0054 |
| Q 3 | 2.67 (2.03, 3.50) | <0.0001 |  | 3.76 (2.07, 6.85) | <0.0001 |
| Q 4 | 4.33 (3.33, 5.63) | <0.0001 |  | 5.26 (2.88, 9.58) | <0.0001 |
| P for trend | <0.0001 |  |  | <0.0001 |  |

**Note:** Model adjusted for age, sex, LDL-C, TC, Scr, AST, ALT, SBP, DBP, drinking, smoking and family history of diabetes

1. **The analysis results based on population with 18.5 kg/m^2^< BMI < 40 kg/m^2^ (Table 4-7)**

**Table 4.** Baseline parameters of population (N = 188,716)

| TyG index | Q1 | | | Q2 | Q3 | | Q4 | P-value | |
| --- | --- | --- | --- | --- | --- | --- | --- | --- | --- |
| Participants | 46265 | | | 47126 | 47456 | | 47869 |  | |
| Age (years, mean ± SD) | 37.94 ± 10.15 | | | 41.11 ± 12.15 | 44.15 ± 13.25 | | 46.71 ± 13.16 | <0.001 | |
| Sex, n (%). |  | | |  |  | |  | <0.001 | |
| Male | 16042 (34.67%) | | | 23768 (50.44%) | 29925 (63.06%) | | 35931 (75.06%) |  | |
| Female | 30223 (65.33%) | | | 23358 (49.56%) | 17531 (36.94%) | | 11938 (24.94%) |  | |
| BMI (kg/m^2^, mean ± SD) | 21.84 ± 2.34 | | | 22.88 ± 2.73 | 24.00 ± 2.97 | | 25.41 ± 3.02 | <0.001 | |
| SBP (mmHg, mean ± SD) | 113.12 ± 14.13 | | | 117.32 ± 15.40 | 121.39 ± 16.11 | | 125.80 ± 16.62 | <0.001 | |
| DBP (mmHg, mean ± SD) | 70.29 ± 9.62 | | | 72.88 ± 10.15 | 75.43 ± 10.56 | | 78.62 ± 10.92 | <0.001 | |
| FPG (mg/dL, mean ± SD) | 83.87 ± 9.28 | | | 87.13 ± 9.48 | 89.81 ± 9.85 | | 93.76 ± 10.47 | <0.001 | |
| TC (mg/dL, mean ± SD) | 166.06 ± 28.74 | | | 176.75 ± 30.96 | 186.82 ± 32.80 | | 198.93 ± 35.01 | <0.001 | |
| TG (mg/dL, mean ± SD) | 52.12 ± 11.72 | | | 81.10 ± 12.38 | 116.79 ± 18.80 | | 203.74 ± 58.75 | <0.001 | |
| LDL-C (mg/dL, mean ± SD) | 79.84 ± 23.34 | | | 85.86 ± 28.01 | 91.15 ± 31.79 | | 95.38 ± 34.69 | <0.001 | |
| HDL-c (mg/dL, mean ± SD) | 53.19 ± 8.78 | | 53.08 ± 8.70 | | 53.12 ± 8.74 | | 53.11 ± 8.74 | | 0.641 |
| ALT (U/L, median (Q1-Q3) | | 14.00 (11.00-19.00) | | 16.60 (12.20-23.60) | | 19.80 (14.30-29.00) | 26.00 (18.00-39.00) | <0.001 | |
| AST (U/L, mean ± SD) | | 21.81 ± 5.97 | | 22.35 ± 6.45 | | 23.04 ± 6.88 | 24.32 ± 8.31 | <0.001 | |
| Scr (mg/dL, mean ± SD) | | 0.74 ± 0.15 | | 0.78 ± 0.16 | | 0.81 ± 0.16 | 0.84 ± 0.16 | <0.001 | |
| Smoker | |  | |  | |  |  | <0.001 | |
| Now | | 1026 (2.22%) | | 2029 (4.31%) | | 3091 (6.51%) | 4569 (9.54%) |  | |
| Once | | 315 (0.68%) | | 534 (1.13%) | | 705 (1.49%) | 805 (1.68%) |  | |
| Never | | 9777 (21.13%) | | 10557 (22.40%) | | 10412 (21.94%) | 9832 (20.54%) |  | |
| Not recorded | | 35147 (75.97%) | | 34006 (72.16%) | | 33248 (70.06%) | 32663 (68.23%) |  | |
| Drinker | |  | |  | |  |  | <0.001 | |
| Now | | 113 (0.24%) | | 237 (0.50%) | | 327 (0.69%) | 544 (1.14%) |  | |
| Once | | 1193 (2.58%) | | 1797 (3.81%) | | 2307 (4.86%) | 2959 (6.18%) |  | |
| Never | | 9812 (21.21%) | | 11086 (23.52%) | | 11574 (24.39%) | 11703 (24.45%) |  | |
| Not recorded | | 35147 (75.97%) | | 34006 (72.16%) | | 33248 (70.06%) | 32663 (68.23%) |  | |
| Family history of diabetes | |  | |  | |  |  | <0.001 | |
| No | | 45433 (98.20%) | | 46111 (97.85%) | | 46450 (97.88%) | 46826 (97.82%) |  | |
| Yes | | 832 (1.80%) | | 1015 (2.15%) | | 1006 (2.12%) | 1043 (2.18%) |  | |

**Table 5.** The results of univariate analysis

|  | Statistics | HR (95% CI) | p |
| --- | --- | --- | --- |
| Age (y) | 42.517 ± 12.687 | 1.064 (1.062, 1.067) | <0.0001 |
| Gender |  |  |  |
| female | 105,666 (55.99%) | Ref |  |
| Female | 83,050 (44.01%) | 0.536 (0.497, 0.577) | <0.0001 |
| BMI (kg/m2) | 23.55 ± 3.08 | 1.246 (1.235, 1.257) | <0.0001 |
| SBP (mmHg) | 119.47 ± 16.30 | 1.038 (1.036, 1.040) | <0.0001 |
| DBP (mmHg) | 74.34 ± 10.78 | 1.045 (1.042, 1.047) | <0.0001 |
| FPG (mg/dL) | 88.68 ± 10.44 | 1.147 (1.143, 1.151) | <0.0001 |
| TC (mg/dl) | 182.29 ± 34.22 | 1.008 (1.007, 1.009) | <0.0001 |
| TG (mg/dL) | 114.08 ± 65.49 | 1.008 (1.007, 1.008) | <0.0001 |
| LDL (mg/dL) | 88.13 ± 30.37 | 1.008 (1.007, 1.009) | <0.0001 |
| HDL-c (mg/dL) | 53.12 ± 8.74 | 1.001 (0.998, 1.005) | 0.4762 |
| TyG | 8.37 ± 0.57 | 5.604 (5.270, 5.959) | <0.0001 |
| ALT (U/L) | 24.09 ± 20.29 | 1.010 (1.009, 1.011) | <0.0001 |
| AST (U/L) | 22.88 ± 7.03 | 1.015 (1.013, 1.017) | <0.0001 |
| Scr (mg/dL) | 0.79 ± 0.17 | 2.48 (2.02, 3.05) | <0.0001 |
| Smoker |  |  |  |
| Now | 10,715 (5.68%) | Ref |  |
| Once | 2,359 (1.25%) | 0.752 (0.565, 1.003) | 0.0522 |
| Never | 40,578 (21.50%) | 0.479 (0.416, 0.551) | <0.0001 |
| Not recorded | 135,064 (71.57%) | 0.643 (0.570, 0.724) | <0.0001 |
| Drinker |  |  |  |
| Now | 1,221 (0.65%) | Ref |  |
| Once | 8,256 (4.37%) | 0.484 (0.334, 0.701) | 0.0001 |
| Never | 44,175 (23.41%) | 0.522 (0.372, 0.733) | 0.0002 |
| Not recorded | 135,064 (71.57%) | 0.566 (0.405, 0.790) | 0.0008 |
| Family history of diabetes |  |  |  |
| No | 184,820 (97.94%) | Ref |  |
| Yes | 3,896 (2.06%) | 1.731 (1.460, 2.053) | <0.0001 |

**Table 6.** Relationship between TyG index and risk of diabetes

| Outcomes | Crude model | |  | Model Ⅰ | |  | Model Ⅱ | |
| --- | --- | --- | --- | --- | --- | --- | --- | --- |
|  | HR (95% CI) | P |  | HR (95% CI) | P |  | HR (95% CI) | P |
| TyG index | 5.60 (5.27, 5.96) | <0.0001 |  | 3.30 (3.08, 3.54) | <0.0001 |  | 3.39 (3.16, 3.65) | <0.0001 |
| TyG (quartile) |  |  |  |  |  |  |  |  |
| Q 1 | Ref |  |  | Ref |  |  | Ref |  |
| Q 2 | 2.83 (2.32, 3.46) | <0.0001 |  | 1.84 (1.50, 2.25) | <0.0001 |  | 1.85 (1.51, 2.26) | <0.0001 |
| Q 3 | 7.00 (5.83, 8.42) | <0.0001 |  | 3.23 (2.68, 3.90) | <0.0001 |  | 3.30 (2.73, 3.99) | <0.0001 |
| Q 4 | 18.23 (15.28, 21.73) | <0.0001 |  | 6.25 (5.21, 7.51) | <0.0001 |  | 6.39 (5.30, 7.70) | <0.0001 |
| P for trend | <0.0001 |  |  | <0.0001 |  |  | <0.0001 |  |

**Crude model:** adjusted for none.

**Model Ⅰ:** adjusted for age, sex and BMI

**Model Ⅱ:** adjusted for age, sex, BMI, LDL-C, TC, Scr, AST, ALT, SBP, DBP, drinking, smoking and family history of diabetes

Table 7. The effect of magnitude of TyG index on diabetes risk stratified by subgroups.

| Characteristics | No. of participants | HR (95%CI) | P -value | P for interaction |
| --- | --- | --- | --- | --- |
| Age (year) |  |  |  | <0.0001 |
| <40 | 96,509 | 4.02 (3.32, 4.86) | <0.0001 |  |
| >=40, <60 | 69,141 | 3.09 (2.78, 3.44) | <0.0001 |  |
| >=60 | 23,066 | 2.37 (2.11, 2.67) | <0.0001 |  |
| Sex |  |  |  | 0.0062 |
| Male | 105,666 | 3.19 (2.92, 3.48) | <0.0001 |  |
| Female | 83,050 | 3.88 (3.40, 4.43) | <0.0001 |  |
| BMI(kg/m^2^) |  |  |  | <0.0001 |
| >18.5, <24 | 111,294 | 4.15 (3.64, 4.73) | <0.0001 |  |
| >=24, <28 | 60,886 | 3.24 (2.91, 3.60) | <0.0001 |  |
| >=28 | 16,536 | 3.09 (2.66, 3.59) | <0.0001 |  |
| SBP(mmHg) |  |  |  | <0.0001 |
| <140 | 169,093 | 3.50 (3.21, 3.81) | <0.0001 |  |
| >=140 | 19,623 | 2.90 (2.54, 3.32) | <0.0001 |  |
| DBP(mmHg) |  |  |  | 0.9985 |
| <90 | 173,322 | 3.31 (3.06, 3.59) | <0.0001 |  |
| >=90 | 15,,393 | 3.48 (2.94, 4.12) | <0.0001 |  |
| Smoker |  |  |  | 0.7188 |
| Now | 10,715 | 3.18 (2.52, 4.01) | <0.0001 |  |
| Once | 2,359 | 4.36 (2.51, 7.57) | <0.0001 |  |
| Never | 40,578 | 3.53 (2.98, 4.17) | <0.0001 |  |
| Not recorded | 135,064 | 3.37 (3.11, 3.66) | <0.0001 |  |
| Drinker |  |  |  | 0.2190 |
| Now | 1,221 | 6.40 (3.03, 13.55) | <0.0001 |  |
| Once | 8,256 | 4.10 (2.90, 5.80) | <0.0001 |  |
| Never | 44,175 | 3.26 (2.82, 3.78) | <0.0001 |  |
| Not recorded | 135,064 | 3.37 (3.10, 3.65) | <0.0001 |  |
| Family history of diabetes |  |  |  | 0.1156 |
| No | 184,820 | 3.42 (3.17, 3.68) | <0.0001 |  |
| Yes | 3,896 | 3.06 (2.09, 4.48) | <0.0001 |  |

**Note 1:** the model was adjusted for sex, age, BMI, LDL-C, TC, Scr, ALT, AST, SBP, DBP, drinking, smoking and family history of diabetes.

**Note 2:** the model was adjusted for all above variables except the corresponding stratification variable.
